# *De novo* transcriptome analysis of halotolerant bacterium *Staphylococcus* sp. strain P-TSB-70 isolated from East coast of India: In search of salt stress tolerant genes

**DOI:** 10.1371/journal.pone.0228199

**Published:** 2020-02-10

**Authors:** Priyanka Das, Bijay Kumar Behera, Soumendranath Chatterjee, Basanta Kumar Das, Trilochan Mohapatra

**Affiliations:** 1 Fishery Resource and Environmental Management Division, Biotechnology Laboratory, ICAR- Central Inland Fisheries Research Institute, Barrackpore, West Bengal, India; 2 Parasitology and Microbiology Research Laboratory, Department of Zoology, University of Burdwan, Burdwan, West Bengal, India; 3 Secretary, DARE and Director General, Indian Council of Agricultural Research, New Delhi, India; ICAR - National Research Center on Plant Biotechnology, INDIA

## Abstract

In the present study, we identified salt stress tolerant genes from the marine bacterium *Staphylococcus* sp. strain P-TSB-70 through transcriptome sequencing. In favour of whole-genome transcriptome profiling of *Staphylococcus* sp. strain P-TSB-70 (GenBank Accn. No. KP117091) which tolerated upto 20% NaCl stress, the strain was cultured in the laboratory condition with 20% NaCl stress. Transcriptome analyses were performed by SOLiD4.0 sequencing technology from which 10280 and 9612 transcripts for control and treated, respectively, were obtained. The coverage per base (CPB) statistics were analyzed for both the samples. Gene ontology (GO) analysis has been categorized at varied graph levels based on three primary ontology studies viz. cellular components, biological processes, and molecular functions. The KEGG analysis of the assembled transcripts using KAAS showed presumed components of metabolic pathways which perhaps implicated in diverse metabolic pathways responsible for salt tolerance viz. glycolysis/gluconeogenesis, oxidative phosphorylation, glutathione metabolism, etc. further involving in salt tolerance. Overall, 90 salt stress tolerant genes were identified as of 186 salt-related transcripts. Several genes have been found executing normally in the TCA cycle pathway, integral membrane proteins, generation of the osmoprotectants, enzymatic pathway associated with salt tolerance. Recognized genes fit diverse groups of salt stress genes *viz*. abc transporter, betaine, sodium antiporter, sodium symporter, trehalose, ectoine, and choline, that belong to different families of genes involved in the pathway of salt stress. The control sample of the bacterium showed elevated high proportion of transcript contigs (29%) while upto 20% salt stress treated sample of the bacterium showed a higher percentage of transcript contigs (31.28%). A total of 1,288 and 1,133 transcript contigs were measured entirely as novel transcript contigs in both control and treated samples, respectively. The structure and function of 10 significant salt stress tolerant genes of *Staphylococcus* sp. have been analyzed in this study. The information acquired in the present study possibly used to recognize and clone the salt stress tolerant genes and support in developing the salt stress-tolerant plant varieties to expand the agricultural productivity in the saline system.

## Introduction

Abiotic stresses pose a considerable risk to agriculture and the environment. Salinity is one of the abiotic ecological issues that badly affect growth, limit yield and geographic distribution of plants [1]. Salinization is one of the significant causes of loss of cultivable land straight adding to the challenge to prolong global food supply. Increasing the yield of staple foods is an absolute requirement for humankind. It is, therefore, essential to produce salt stress-tolerant plants for farming of saline lands [2]. In this context, a need arises to employ genetic techniques to develop crops in the coming decades. Prokaryotes can stay alive in a broad assortment of natural stresses, like extremes of temperature, pressure, salinity, pH, radiation, etc. [3]. Halophilic and halotolerant bacteria dwell in a broad base of salty habitation. Moderately halotolerant bacteria are much more dispersed than excessive halotolerant microbes [4]. Competence of the bacteria for responding and maintaining in fluctuations of external osmolarity is a striking function for survival and proliferation in varied environmental niches [5] acquired during evolution through the expression of a broad spectrum of salt stress tolerant genes. Therefore, it is of utmost important to search for the elite and fastidious organisms, including bacteria compassing capable genes to augment agricultural production.

Extreme salt habitats are dynamic and unique surroundings which offer tremendous prospects to increase understanding of genes responsible for salt tolerance and hypersaline cellular physiology [6]. The diverse kind of halophilic and halotolerant microorganisms have been recognized from an ample range of aquatic atmosphere [7–11]. Halophilic and halotolerant bacteria are a dweller of saline environments and mixed in propagation. The moderately halotolerant microorganisms are more disseminated compared to extremely halotolerant microorganisms [4]. These elite classes of bacteria have developed distinctive properties throughout the evolution to adjust to the altering ecological condition viz. salinity that acquires intrinsic competence to bear with the hypersaline environment [11]. These exclusive properties are demonstrated unforeseen owing to the progression of evolution as well as the expression of a broad range of salt stress tolerant genes. Many salt stress tolerant genes like stpA [12]; betS [13]; OtsBA [14–15]; ectABC [16–18] have been identified globally. The next-generation RNA sequencing technology is in use extensively in comparative transcriptomics for the identification of alterations in transcript abundance among altered treatment conditions [19–20]. Numerous tools have been established to infer and enumerate the transcriptome, *viz*. hybridization technique and sequence-based approaches. These approaches have various limits, *viz*: dependence upon current information about genome sequence and an incomplete dynamic variety of finding due to both background and saturation of signals [21]. Recent RNA sequencing by way of high-throughput technology has modernized the study of the microbial transcriptome [22–24]. Several researchers have studied the differential gene expression of several bacteria in different stressed conditions through transcriptome profiling since RNA-seq analysis has proved the advantages over the disadvantages through hybridization technique and sequence-based technique [25–30].

Whole-genome transcriptome analysis has become very useful for the detection and characterization of candidates and novel genes and to understand their functions [31]. *Staphylococcus* sp. is a Gram-positive, mostly non-pathogenic bacteria fitting into the family Staphylococcaceae found worldwide, which is a small constituent of soil microbial flora [32].The genus *Staphylococcus* consists of 47 different type species and 24 different subspecies with dependably accessible names [27]. According to Choi and co-workers, resources for isolation of *Staphylococcus* species comprise of amber [33], insects [34], estuarine and aquatic surface water [35], fermented fish [36], cheese [37], soil, and plant, signifying pervasiveness of the *Staphylococcus* in the normal environment, while, ecology and physiology of non-pathogenic *Staphylococcus* in the natural habitat resided ambiguously. A general characteristic of *Staphylococus* species is tolerant to high NaCl concentration [27]. Similarly, high salt stress-tolerant *Staphylococcus* was perceived in a variety of experiments [38]. *S*. *condimentii*, *S*. *piscifermentans*, and *S*. *carnosus* can propagate at 15% NaCl [27] and *S*. *agnetis* can tolerate even upto 19% NaCl [39]. *Staphylococcus* sp. is unsurprising to experience varying ecological surroundings and stresses, as well as osmotic stress as *Staphylococcus* sp. subsists in different environments that include outside the animal, food, natural environment, and human host. Ming-xiang et al. [40] studied the molecular epidemiological characteristics and antimicrobial resistance of clinical isolates of *Staphylococcus aureus* in the Changsha area detailing that the strain was resistant to penicillin G, ampicillin, erythromycin, and clindamycin.

Large scale data generated in this study would place an excellent foundation for further research into the novel gene discovery, inheritances, genomics, as well as, proteomics of *Staphylococcus* sp. Furthermore, the transcriptome sequences acquired in this study provide the conscious of the genetic constitution of the microorganisms and the sequences created here are convenient for additional research into salt tolerance.

Numerous researchers have deliberated on the differential gene expression of quite a lot of bacteria in altered stress conditions with the help of transcriptome profiling since RNA-seq analysis has evidenced merits over the demerits through hybridization technique and sequence-based technique as well. Scanty literature is available related to the isolation of salt stress tolerant bacteria having salt stress tolerant genes along the East coast of India. Several studies have been performed by researchers for identification of salt stress tolerant genes from bacteria and as well as several other prokaryotes and eukaryotes through microarray analysis. Through in current years, there is an increase in the number of bacterial transcriptomics data addition in the existing databases, the description of novel genes and concerned respective biological functions have also been found individually. Nonetheless, a lot of research study is essential till date to mine genes and alleles from diverse bacteria bearing salt stress tolerant gene property.

In this backdrop, the transcriptome dataset has been generated to furnish genetic information to discover the salt stress tolerance mechanisms using the SOLiD platform. The transcriptomes of control and salt-stressed bacteria have been compared to recognize genes screening transcriptional changes, to recognize the functions of the transcripts, to identify the significant biological pathways that explained the changes and the novel transcripts which could be associated with salt stress.

## Materials and methods

### RNA isolation, transcriptome library preparation, and SOLiD sequencing

**Fig 1:**
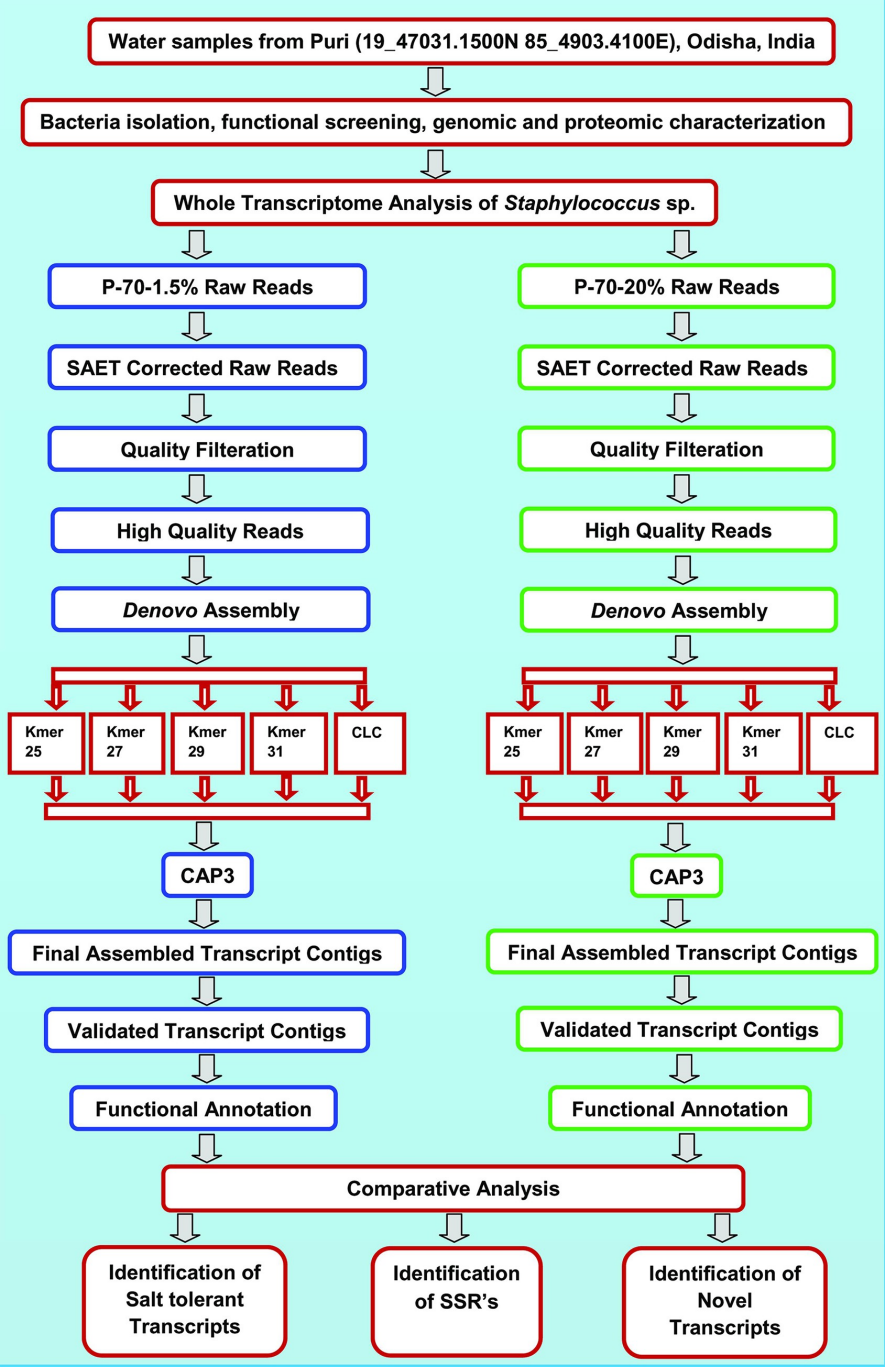
Schematic workflow of the methodologies of transcriptome analysis of both control and treated bacterial samples.

The total schematic workflow of the methodology for both control and treated samples has been given in Fig 1. Total RNA was extracted from the overnight grown cell cultures for both control (1.5% NaCl) and treated (20%NaCl) samples of the bacterial isolate in TSB medium (HiMedia Pvt. Ltd., Mumbai) with RaFlex total RNA isolation kit following manufacturer’s protocol. Xcelgen plant RNA isolation kit was used in combination with the removal of genomic DNA contamination. Total RNA (1 μg) was analyzed on 1% denaturing agarose gel. Both the group of samples (control and treated) were run in triplicate for transcriptome analysis.

**Fig 2:**
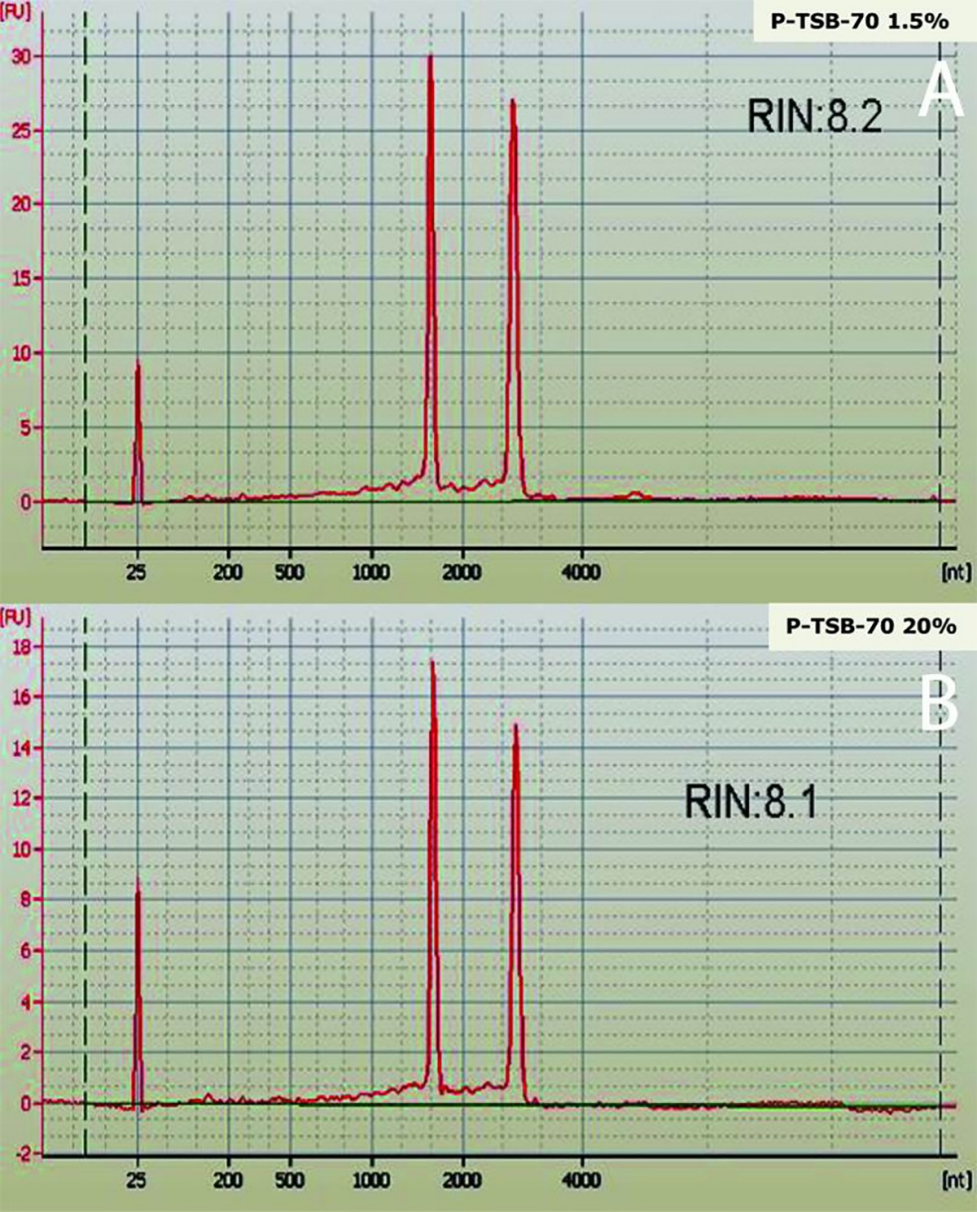
Bioanalyzer profiles of total RNA of A) P-TSB-70_1.5% control sample and B) P-TSB-70_20% Treated sample on Pico 6000 chip.

Both the RNA quality as well as the RNA quantity, was identified by Agilent 2100 BioAnalyzer (Agilent Technologies, CA, USA) (Fig 2).Total RNA of both control and treated bacterial samples was used to enrich the RNA transcripts by selectively depleting ribosomal RNA molecules (rRNA) using the Ribominus bacteria module (Invitrogen). For transcriptome library preparations, RNA samples from both the groups were set using the SOLiD Total RNA Seq kit following the manufacture’s protocol. For library amplification, the size selection and in-gel PCR was executed [41]. The amplification was reviewed on 2% agarose gel. A high sensitivity DNA chip was used to quantify the transcriptome library using Bioanalyzer. The template bead for both the library was prepared, and clonal amplification of the library was carried out using Emulsion PCR. Post-Emulsion PCR, bead wash was carried out and template beads enrichment was executed. Alteration of 3' ends was executed for deposition of beads on a glass slide. The Nanodrop spectrophotometer was used for bead quantification. A workflow study was performed on the SOLiD Analyzer for beads quality confirmation. The sequencing run was performed using SOLiD TOP SEQ-FRAG LIB KIT MM 50 by outsourcing (Xcelris Pvt. Ltd.). The sequencing of both bacterial samples was performed using a SOLiD 4.0 Genome Analyzer for long paired-end reads generation. The data acquired was submitted to NCBI SRA submission portal database with a series of Accession Number PRJNA509749.

### Transcriptome assembly

The acquired raw data were filtered through the standard SOLiD pipeline. The high-quality filtered reads received after initial screening were used for further downstream processing. After stringent filtering, high-quality reads of every sample were subjected to assembly.

For optimal assembly generation of the bacterial transcriptome, the assembly was carried out with all the high-quality reads (after removal of duplicate reads from both the samples). The average probability of error for base calling was found low (<0.2%) in the first 59 sites and advanced to 2% at the end site. The raw reads were SAET corrected, followed by quality filtration. The high-quality reads of both the control and treated samples were assembled with the de novo assembly pipeline using Velvet v1.2.01 followed by Oases v0.2.04 to construct unique consensus sequences [42]. De novo assembly was accomplished with different odd *k*-mers for each sample (*k*-mer 25, 27, 29 and 31). Combined transcripts from the various k-mer assemblies were run throughout the CD-HIT-EST program for the removal of redundant transcripts sharing 100% identity [43]. Separately, CLC Genomics Workbench v4.7.2 was also used to carry out the de novo assembly. Ultimately, all the transcript contigs created via Velvet/Oases pipeline and CLC genomics workbench were merged as well as the redundant ones were removed. In the end, longer transcript contigs were generated using the CAP3 assembler program. The eminence of assemblies created was considered via the Perl scripts specified in the NGS QC Toolkit. The mapping of these transcript contigs was done on the high quality reads for removal of misassembled transcript contigs. At least 3 mapped reads per transcript contig was set as criteria for validation of transcript contigs.

### Functional annotation of the assembled transcript contigs

The alignment of the transcript contigs was performed to NCBI non-redundant database of bacteria (http://www.ncbi.nlm.nih.gov/) with the BLASTX program for functional annotation. Both the assemblies were further annotated with BLAST2GO software [44] and also used for the RNA-Seq mapping for differential expression analysis.

### Gene Ontology (GO) mapping, analysis, and distribution

GO mapping was carried out to facilitate recover GO requisites in support of all the BLASTX functionally interpreted transcript contigs. Accession IDs acquired from BLASTX results are used to regain gene names or symbols. Accession IDs of BLASTX result facilitated to regain UniProt IDs aided with PIR which comprise various databases viz. UniProt, PSD, RefSeq, GenPept, PDB, TrEMBL and SwissProt.

The functions of the predicted transcript contigs were classified using GO assignments. The Accession IDs obtained from the BLASTx result aided to recover gene names or symbols. The gene names or symbols thus identified were subsequently investigated in the species-specific accesses found in gene-product tables available in the GO database. GO mapping resulted in the recovery of GO terms for annotated transcript contigs using diverse databases. The total number of GO terms associated with BLASTx hits were established in the UniProtKB database for *Staphylococcus* sp. control and treated transcript contigs.

GO has been categorized at different graph levels on account of three main ontologies: molecular functions, biological processes as well as cellular components. The initial levels signify the common functions of transcript contigs. As the levels progress, the functions of the transcript contigs become more significant. GO annotations were assigned using the QuickGO (http://www.ebi.ac.uk/QuickGO/) tool accessible at EBI [45]. After GO term enrichment, the annotated transcripts of both the samples were additionally annotated through InterPro (http://www.ebi.ac.uk/interpro/) tool [46].

### Pathway enrichment analysis with KEGG

The current knowledge of biochemical pathways and other types of molecular interactions has been computerized with the help of KEGG, which can be used as a reference for the systematic understanding of sequence data [47]. Besides GO term annotation, the transcripts of both control and treated sample were annotated using KO by KAAS (http://www.genome.jp/tools/kaas/) [48]. The transcripts were analyzed with the Bidirectional Best Hit method to acquire the KO conditions for both the transcripts with a BLAST threshold of 40.

### Identification of salt-tolerant and novel transcripts

The annotated transcript contigs of control and treated samples were further used for the identification of salt-tolerant transcript contigs. Both control and treated annotated transcripts contigs were subjected to comparative genome-based study for identification of salt-tolerant and novel transcripts.

### Heat map

An average linkage hierarchical cluster analysis was executed, leading 100 differentially expressed genes with Multiple experiment viewer (MEV v4.9.0). Expression levels are characterized by the log2 ratio of transcript profusion among control and treated samples. Differentially expressed transcripts recognized in control and treated conditions were analyzed through hierarchical clustering. The log-transformed and normalized value of transcripts was used to generate a heat map derived from Pearson correlation distance that is derived from the average linkage method [49, 30].

### Protein structure prediction

Identified salt stress-tolerant/related gene transcripts were considered for functional protein structure prediction utilizing open reading frame analysis through ORF Finder (https://www.ncbi.nlm.nih.gov/orffinder/). Only full-length protein sequences were considered for molecular modelling. Ten salt-tolerant genes were chosen with a full-length coding sequence (CDS). Predicted full-length CDS were then considered as target protein sequences in the protein modelling pipeline. A homology modelling approach was carried out to generate the predicted 3 dimensional (3D) structure of salt-stress tolerant functional genes (five) as well as the genes related to salt stress mechanism (five). A BLAST-P analysis against PDB was carried out to identify the suitable template for homology modelling exercises. Altogether ten protein structures were generated considering the models with lowest DOPE Score (discrete optimized protein energy) and least restraints violations using MODELLER 9.11 (https://salilab.org/modeller/9.11/release.html) software. The probability density functions (pdfs) were utilized by MODELLER software in preference to energy as spatial restraints [50]. The generated structural co-ordinate files were validated through Ramachandran plot analysis (http://services.mbi.ucla.edu/PROCHECK) [51]. The structural quality was also measured by analyzing their Z-score using the ProSA-web server [52, 53]. The structural details (helix, turn, strand, H-bond) were calculated using RasMol (http://www.openrasmol.org/) [54].

## Results

### Transcriptome assembly

CAP3 assembly resulted in 11796 and 10973 transcript contigs for control and treated samples respectively with transcript contig N50 value greater than 200 for both the samples. The maximum length of the transcript contig was found to be 2.3 kb and 2.5 kb for control and treated sample, respectively. Both the control and treated transcriptome assemblies were found to be comparable based on the assembly statistics. The mapping of all high-quality reads recommended that during the final combination assembly, 95% of reads were used and the average read depth of every contig was 196 and 208, respectively, for both control and treated sample. Furthermore, comparative analysis from assembly results of 50-bp to 80-bp reads illustrated that with the increase in the length of the reads, the number of assembled sequences, the N50 value and the average length of the sequences visibly increased. The N50 value for control and treated are 204 and 228, respectively. The average coverage per base statistics has been given in S1 Table.

### Annotated data distribution

For the *Staphylococcus* sp. control sample BLASTx annotated 10,280 transcript contigs out of 11,796 while 1,516 transcript contigs had no significant BLAST hits. These annotated transcript contigs were mapped on the GO database, out of which 6,174 were annotated through the GO database whereas 1,684 were found unassigned to any GO terms. Similarly for the *Staphylococcus* sp. treated sample BLASTx annotated 9,612 transcript contigs out of 10,973 while 1,361 transcript contigs had no significant BLAST hits. These annotated transcript contigs were mapped on the GO database out of which 6,192 were annotated through the GO database whereas 1,468 were found unassigned to any GO terms (S1 Fig).

BLASTx hits for every transcript contigs lie within the cut-off value 1e-06 and high significant similarity was shown in the transcript contigs. The EC sharing for sequences and BLASTx hits graphs indicate how EC, which represents experimental evidence for the existence of protein, is associated with the obtained GO term and its annotation. Similarity distribution showed that around 90% of transcript contigs had constructive alignment length between 50–90% in both control and treated samples. An optimal annotation score was attained for every transcript contigs through the minimum similarity of 55% and above. The maximum number of transcript contigs annotated was between 65–70% similarities for both samples. The annotation distribution graph showed the relation between the sequences and the number of GO terms with those they were annotated. A significant number of transcript contigs were annotated with 1 to 5 GO-levels for both bacterial samples transcript contigs.

### Gene ontology level and sequence distribution

GO has been categorized at different graph levels based on three significant ontologies: molecular functions, biological processes, and cellular components. In the case of control and treated samples, cellular components were found to be maximum at 5th level and molecular functions at 7th GO-level whereas for biological processes it was at 6th GO-level (S2 Fig). Gene ontology distribution can be used for a better understanding of the allocation of annotated transcript contigs in specific ontology domains, for instance, molecular function, cellular component or biological process. From the full amount of annotated transcript contigs, 10,280 and 9,612 transcript contigs were mapped to GO for control and treated respectively. The highest numbers of transcript contigs were allowed to molecular functions. One transcript contigs can be categorized into more than one GO domain. GO sequence distributions, facilitates in identifying entire annotated nodes consisting of GO functional groups. Transcripts allied with related functions are allocated to the same GO functional group. The GO sequence sharing was investigated for all the three gene ontologies.

"Metabolic process" is the primary biological process in both the bacterial samples as ~85% of the transcript contigs in the samples are related to this ontology, which involves cellular metabolic processes, macromolecular metabolism, and nitrogen metabolism. Approximately, 80% of transcript contigs were also found to be related to "cellular process", involving cellular, response to the stimulus, cellular localization, DNA conformation change, and cellular developmental process, whereas 30% was associated with the 'Response to stimulus" ontology that includes feedback to abiotic and biotic stresses, chemical stimuli, temperature, and inorganic stimuli.

"Cell" is the main cellular component in both the bacterial samples which constitutes about 98% of the total transcript contigs. This includes the intracellular component, the membrane component and the envelope component among many others. "Binding activity" is the main molecular function in both the bacterial samples as around 62% of the transcript contigs in the samples were related to this ontology. The "binding activity" includes binding to ion, protein, lipid, carbohydrate, nucleotide & nucleic-acid, co-factor, vitamin, amine, chromatin binding, etc. Around 63% of transcript contigs were responsible for the "catalytic activity" that includes oxido reductase activity, transferase activity, kinase activity, hydrolase activity, and ligase activity, etc.

### Comparative analysis of *Staphylococcus* sp. sequence distribution based on gene ontology study

**Fig 3:**
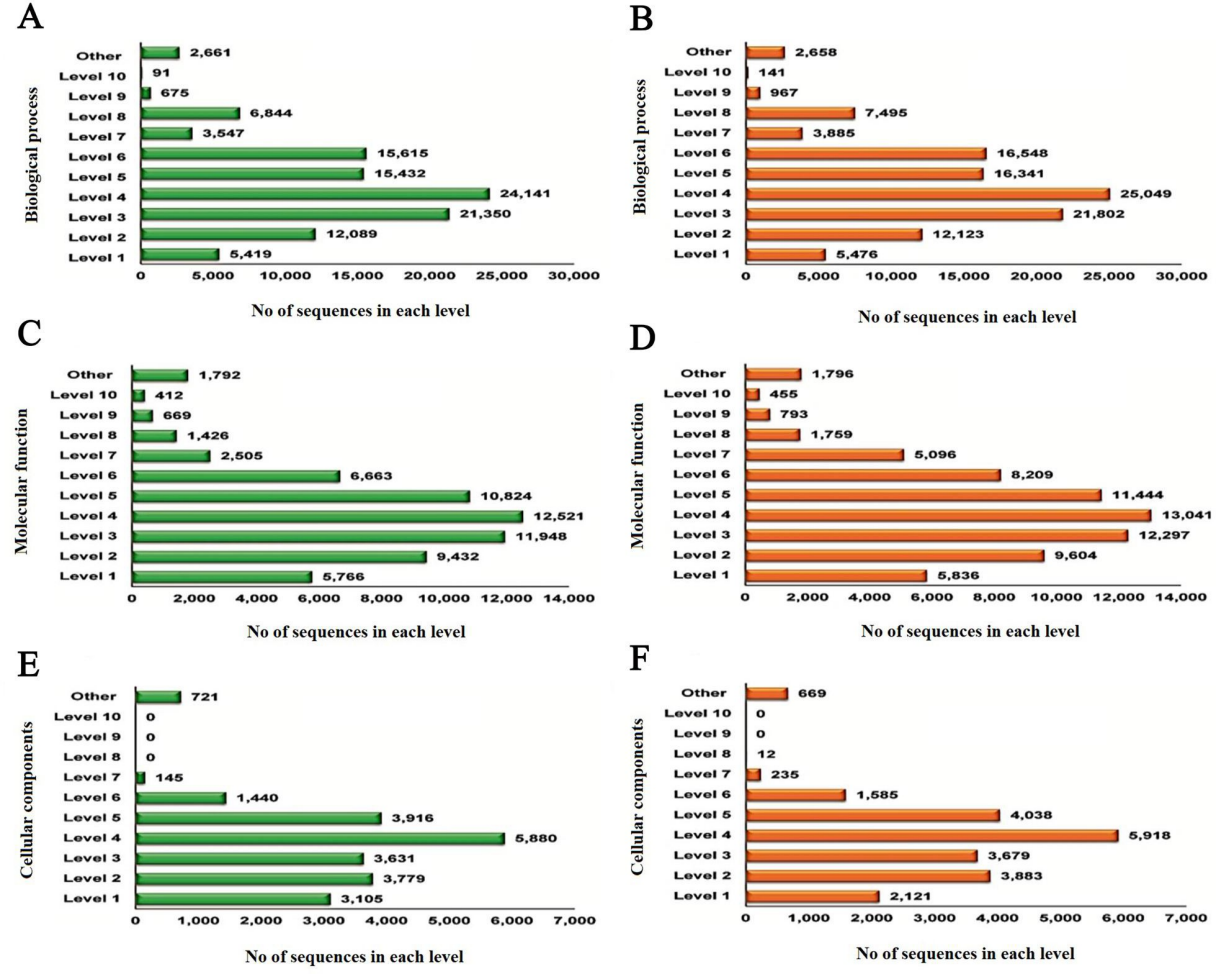
GO Level distribution of both control (A, C, E) and treated (B, D, F) transcripts in three domains.

GO terms were clustered into diverse levels for all the three gene ontologies. The primary levels indicate the universal function of transcript contigs. As the levels progress, the role of the transcript contigs becomes more significant. Every transcript contig can be multifunctional and consequently, can have more than a single role. As a result, the number of transcript contigs for every level differs extensively as shown in Fig 3. For instance in Fig 3, if the biological process is considered at GO level 4, there are a total of 24,141 and 25,049 transcript contigs for all the functions involved in that level for the control (A) and treated (B) sample, respectively. Likewise, for molecular function at GO level 4, there were a total of 12,521 and 13,041 transcript contigs for the control (C) and treated (D) sample and for cellular components at GO level 4, there were 5,880 and 5,918 transcript contigs for all the functions involved in that level for the control (E) and (F) sample, respectively. However, the figure of annotated transcript contigs for the two samples was only 10,280 and 9,612. These shows there are definite transcript contigs that are implicated in more than one function in both the samples.

**Fig 4:**
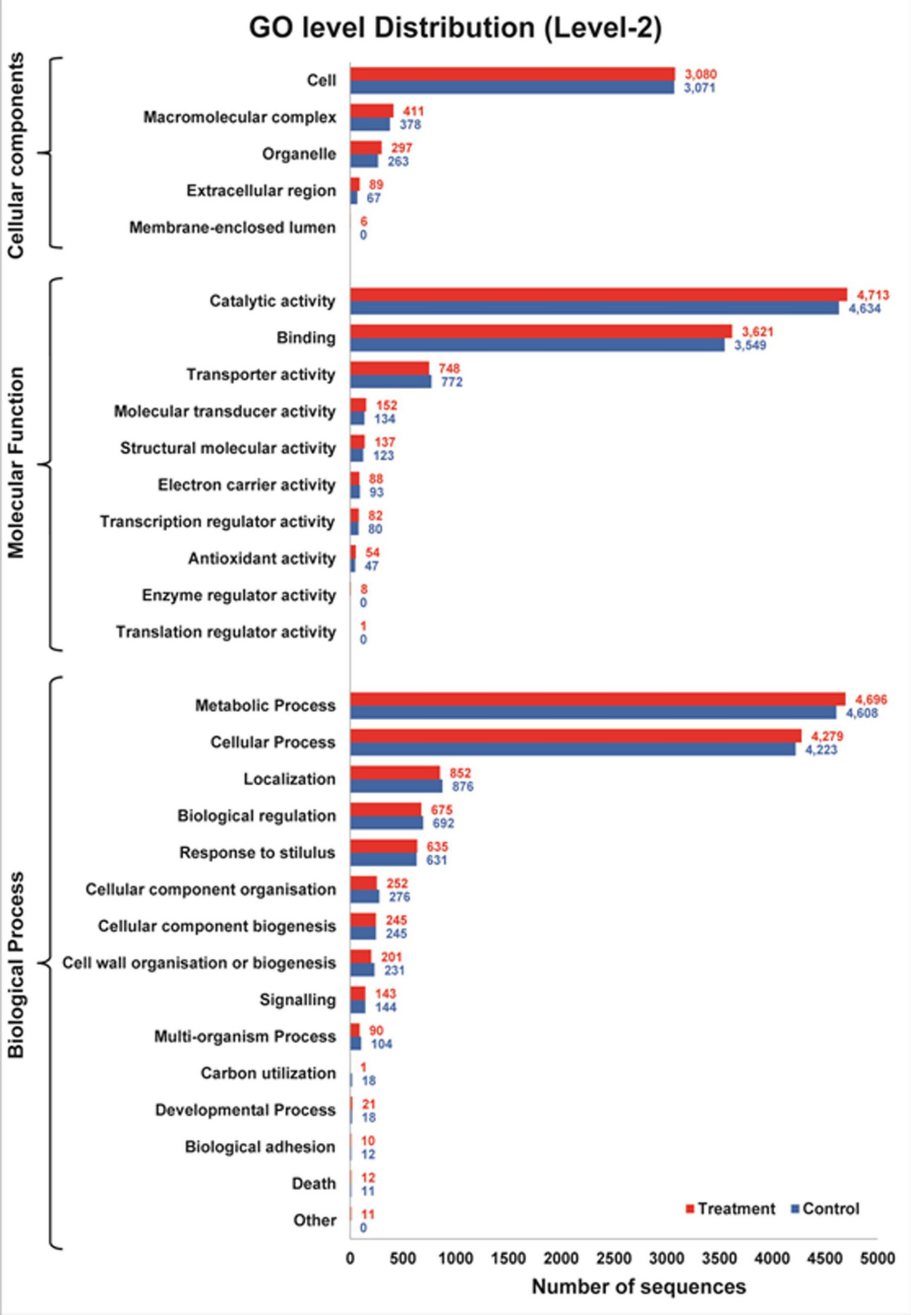
GO Level 2distribution of both control and treated transcripts in three domains.

**Fig 5:**
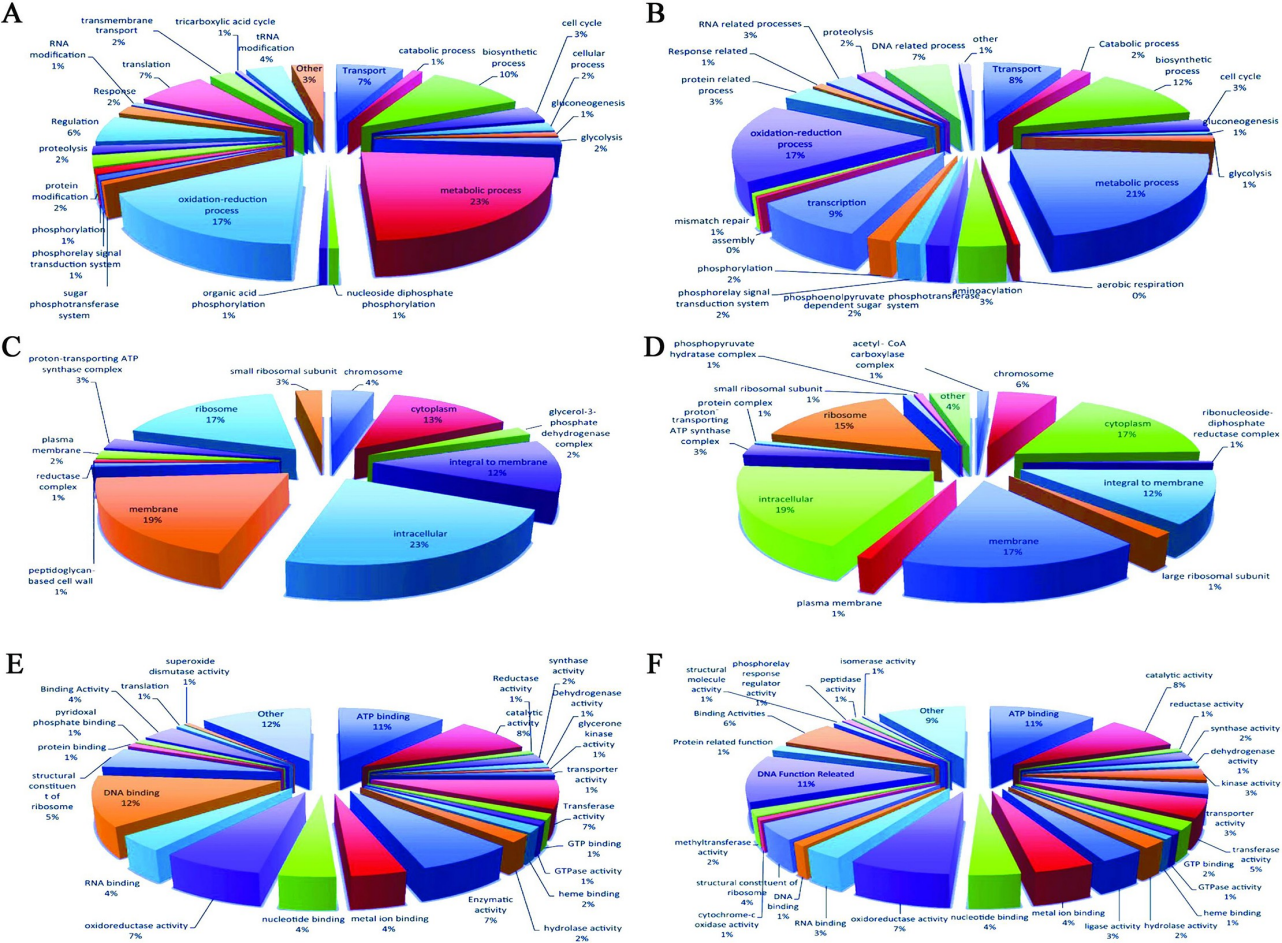
Pie diagram of (A) control transcripts involved in biological processes in GO study (B) treated transcripts involved in biological processes in GO study (C) control transcripts involved in molecular functions in GO study, (D) treated transcripts involved in molecular functions in GO study, (E) control transcripts involved in cellular components in GO study, (F) treated transcripts involved in cellular components in GO study.

In GO level 2, cellular components showed five different domains of distributions *viz*. cell, organelle, membrane-enclosed lumen, extracellular region, and macromolecular complex. Molecular functions showed ten different areas of dispositions *viz*. transcription regulatory action, transporter activity, binding action, catalytic action, molecular transducer activity, structural molecular activity, electron carrier action, antioxidant activity, enzyme regulatory activity as well as translation regulatory activity. Biological processes showed 15 different domain of distributions *viz*. localization, metabolic process, cellular component organization, response to stimulus, cellular process, biological regulation, carbon utilization, cellular component organization, cellular component biogenesis, cell wall organization of biogenesis, signaling, multi-organism process, developmental process, biological adhesion death and other. The number of sequences varied from a maximum of 4713 number of sequences in treated compared to 4634 number of sequences in control in molecular functions while the minimum no of sequences were found to be one in treated and zero in control in molecular functions. The GO level 2 distribution of both control and treated transcripts was illustrated in the graph (Fig 4). The GO Level distribution of both control and treated transcripts of three domains were shown in Fig 5. The number of sequences varies amid in both control and treated conditions in case of other domains of the GO distribution. The pie diagram of all the transcripts of both control and treated sample in three GO studies are shown in the pie diagram (Fig 5).

### Gene ontology analysis at the protein level and metabolic pathway enrichment

From the significant hits obtained from BLASTx results of the transcript contigs a total of 19892 sequences (10280 transcripts of control and 9612 of treated) were conceptually translated using Transeq (http://www.ebi.ac.uk/Tools/st/emboss_transeq/) from EMBOSS in all three forward and three reverse frames i.e., in all six frames. The most extended reading frames were calculated for all the sequences with the help of a custom Perl script developed in house. The InterProScan searches revealed a total 1719 GO term for control transcript and 6797 GO terms for treated transcript. Out of the 10280 control transcripts, 1719 GO terms were distributed into 1319 biological processes, 431 cellular components, and 1720 molecular functions. In the case of treated transcripts, a total of 6797 GO terms were possible to assign to 9612 sequences. Theses GO terms correspond to 4706 biological processes, 1534 cellular components, and 6797 molecular functions. Besides, InterProScan also resulted in a total of 1921 functional domain markers (FDMs) for control transcripts, whereas 7723 DMS for treated transcripts.

The KEGG analysis KAAS of the assembled transcripts exposed presumed representatives of metabolism pathways which perhaps concerned with diverse metabolic pathways that are further involved in salt tolerance. Pathway enrichment using the KAAS server was able to find 10280 sequences associated with 212 KEGG pathways in control transcript contigs and 968 KEGG pathways related to 9612 sequences in treated transcript contigs. Moreover, it was also observed that transcripts from both the samples were involved in some common pathways responsible for salt tolerance including glycolysis/gluconeogenesis, oxidative phosphorylation, glutathione metabolism, etc.

### Identification of salt-tolerant transcript contigs in *Staphylococcus* sp. control and treated sample

The annotated transcript contigs of control and treated samples were further used for the identification of salt-tolerant transcript contigs. Out of 10,280 annotated control transcript contigs, a total of 161 salt-tolerant transcript contigs were identified exclusively in the control sample (S3 Fig). Similarly, out of 9,612 annotated treated transcript contigs, a total of 186 salt-tolerant transcript contigs were identified solely in the treated sample (S3 Fig). The Venn diagram (S4 Fig) represents the316 standard unique salt transcript contig annotations that represent 991 control and 992 treated transcript contigs, respectively, 115 individual salt transcript contig annotations that represent 161 transcript contigs present exclusively in the control sample and 120 individual salt transcript contig annotations that represent 186 transcript contigs present solely in the treated sample. In response to salt stress, out of 186 transcripts, only 90 salt stress-related genes were found to have significant similarity with the current non-redundant NCBI gene database. All these 90 genes belong to diverse groups of salt stress tolerant genes viz. abc transporter (viz. *mlaE*, *modA*, *modB*, *modC*, *opuBA*, *gltJ*), glycine betaine (viz. *betB*, *opuCB*, *opuBB*, *propP*), sodium antiporter (viz. *citT*, *atpH*, *atpC*, *mrpC*, *nhaA*, *nupG*), sodium symporter (viz. *pupP*, *sstT*, *gltT*, *gltS*), and trehalose (viz. *treZ*), which be in different families of genes concerned in the pathway of salt stress. The 90 genes have been further classified in their respective subgroups which have been depicted below in the tabulated form. All these 90 genes have a sum of 344 different similar target gene contigs of the individual gene. Among them, all the 54 different abc transporter genes (viz. *nhaE*, no*dA*, *nodB*, *nodC*, *opuBA*, *gltJ*) have 223 different sequences similar to functional target genes; all the 11 betaine related genes (viz. *betB*, *opuCB*, *opuBB*, *proP*) have 53 different sequences similar to functional target genes; all the eight sodium antiporter genes have (viz. *citT*, *atpH*, *atpC*, *nppC*, *nhaA*, *nupG*) 39 different sequences similar to functional target genes; all the 15 sodium symporter genes (viz. *putP*, *sstT*, *gltS*, *setA*, *gltT*, *acpP*, *sdcS*) have 27 different sequences similar to functional target genes; and the two trehalose genes have two sequences similar to functional target genes. From the findings, it was noticed that all of the specific salt stress genes have numerous sequences that are similar to the respective functional target genes in the genome of *Staphylococcus* sp. bacteria. Moreover, GO study confirms that all the 90 genes were found under some common functional annotated gene clusters. Based on the functional similarity, all the genes were classified among ten different groups. The biological processes, cell components and molecular functions of all the 10 groups have been documented from the gene ontology studies. Besides, all the genes have been found up-regulated in one, two or more times during salt stress. The summaries of 90 genes with high expression levels in response to elevated salt stress have been documented in the tabulated form (S2–S6 Tables). Several other genes have also been found which showed both upregulation and downregulation due to salt stress. Most of those genes belong to the glycolytic cycle pathway, tricarboxylic acid cycle pathway, metal ion transport pathway, different other transport-associated proteins, and different membrane transport pathway associated proteins related genes. The classification of the gene ontology study of the functionally annotated genes has been documented (S7 Table). The transcripts were screened in the UniProtKB protein database to confirm the functional pathway of the respective genes along with the gene name. The genes with the pathways they are involved in have been documented. The list of upregulated genes associated with salt-tolerant pathways as well as other associated metabolic pathways has been recorded (S8 and S9 Tables).

### Identification of novel transcript contigs in *Staphylococcus* sp. control and treated sample

Based on the functional annotation results with BLASTx a total of 1,516 and 1,361 transcript contigs in control and treated sample was found to have no significant blast hits. Sequence similarity search using pairwise alignment by blastn was performed on these transcript contigs of control and treated sample to identify the similar transcript contigs present in both the samples. As a result, 228 transcript contigs showed alignments while 1,288 and 1,133 transcript contigs did not align at all and were considered exclusively as novel transcript contigs in control and treated sample respectively (S5 Fig).

### Gene expression profiling of control and treated samples

A total of 3213 denovo contigs for control and 3447 *de novo* assembled contigs for treated samples having a size greater than 200 bp were considered for differential gene expression (DGE) analysis. The DGE was accomplished using NoiSeq (R package). Differential gene expression study includes all the genes of the cell, including housekeeping genes and the genes responsible for stresses and non-stresses.

### Heat map

**Fig 6:**
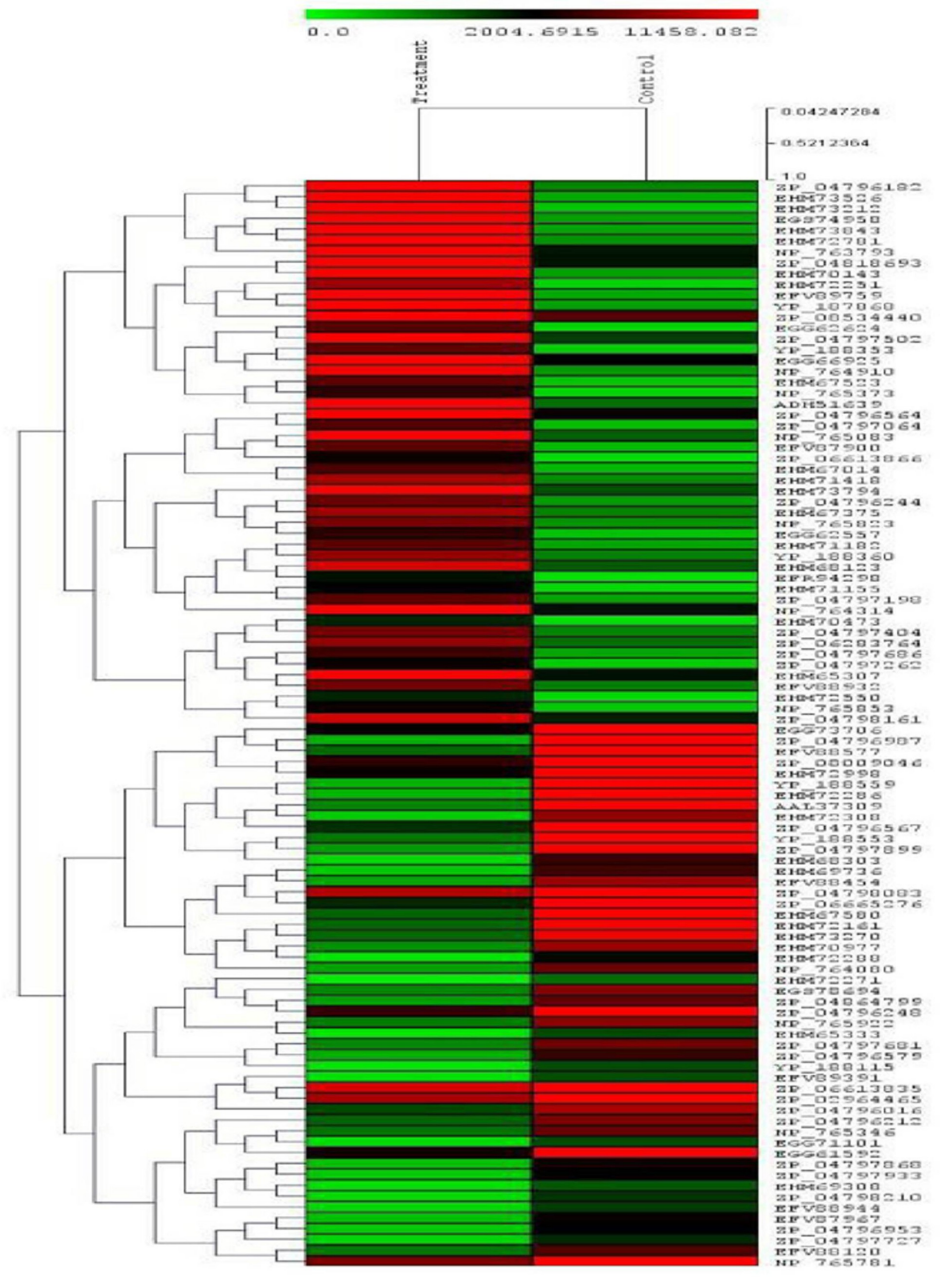
Heat map (cluster) showing the top 100 differentially expressed genes.

Heat map (cluster) generated based on the average linkage hierarchical cluster analysis shows the level of transcript abundance of the top 100 differentially expressed transcripts recognized in control and treated conditions. The heat map depicts that out of 100 genes, almost 50% of the genes were shown to be upregulated, and the rest 50% approximately to be down-regulated. Among 100 genes, candidate salt stress tolerant genes and the genes responsible for other biochemical as well as metabolic pathways are present (Fig 6). The red and green colors in the heat map represent upregulated and downregulated genes, respectively, among the top 100 differentially expressed transcripts in both control and treated conditions.

### Protein structure prediction

**Fig 7:**
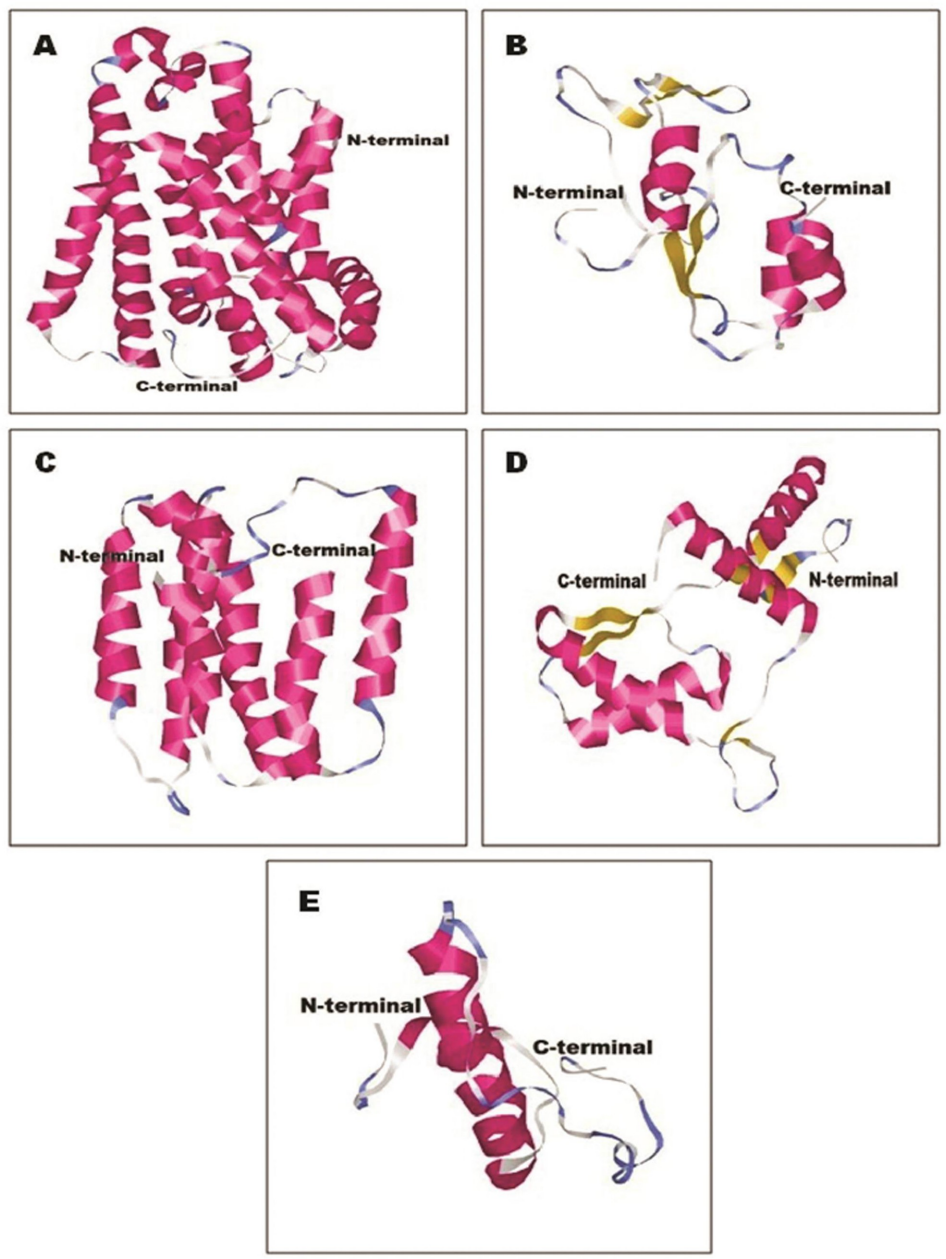
3D structural information of salt tolerance functional genes showing N and C-Terminal, where (A) designated with treatment_transcript_1371-betaine carnitine choline family, (B) treatment_transcript_1069-bacterial abc transporter protein, (C) treatment_transcript_6679-proline betaine transporter, (D) treatment_transcript_4687-betaine aldehydede hydrogenase and (E) treatment_transcript_1432-dass family.

**Fig 8:**
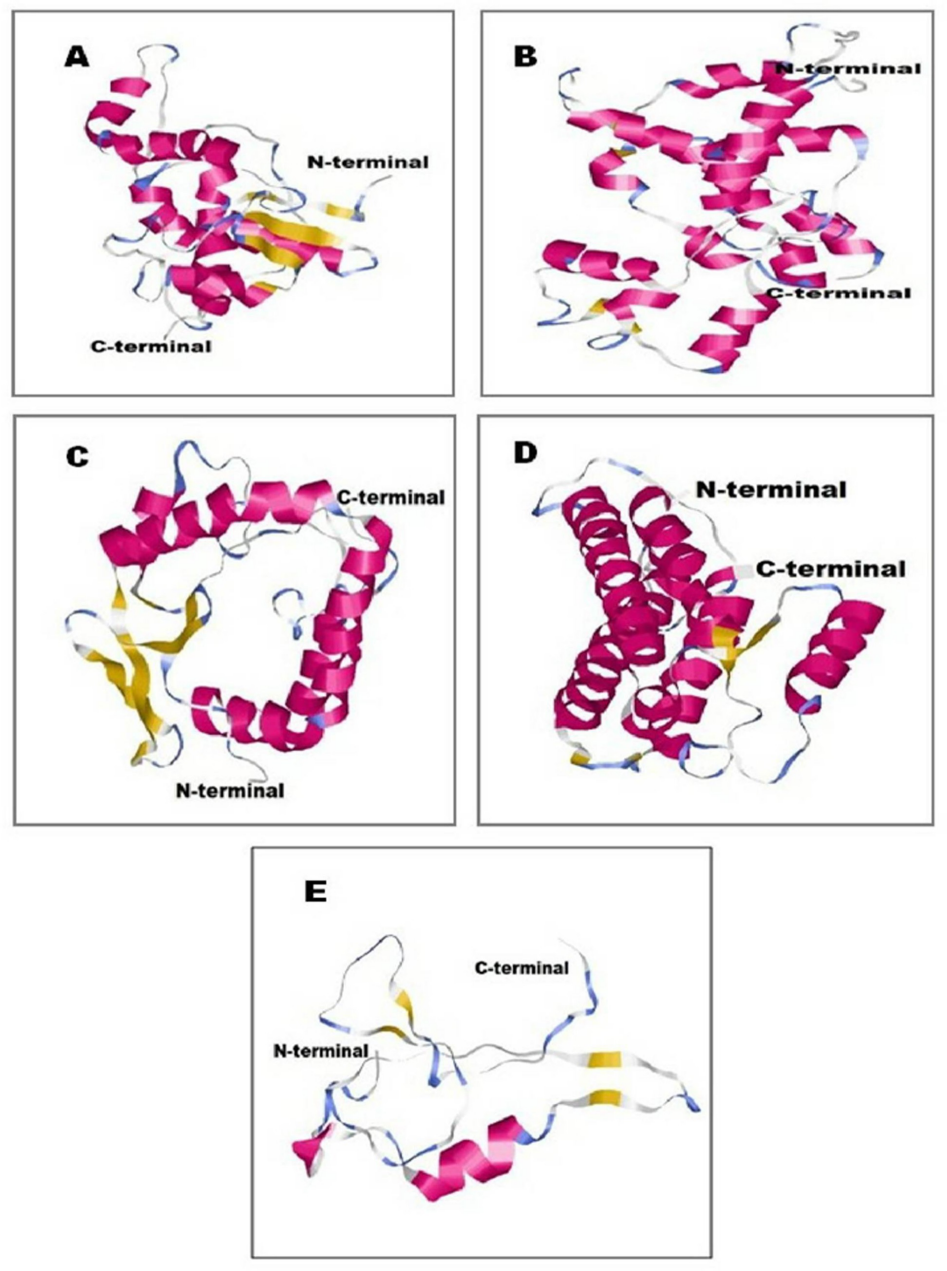
3D structural information of salt tolerance related functional genes showing N and C-Terminal, where (A) treatment_transcript_194-succinate dehydrogenase iron-sulfur subunit, (B) treatment_transcript_5134-atp synthase gamma subunit, (C) treatment_transcript_6954-succinate dehydrogenase flavoprotein subunit, (D) treatment_transcript_7764-succinate semialdehyde dehydrogenase and (E) treatment_transcript_10031- fumarate class ii.

3D structure of salt-tolerant proteins was generated considering the lowest DOPE score for 10 (salt stress-tolerant and associated) genes (Figs 7 and 8). The DOPE score was found highly negative for all the native protein structures. The structural coordinate files were validated by Ramachandran plot analysis. Ramachandran plot analysis revealed that maximum amino acid residues of the protein structures were in their most favourable zone. The protein quality analysis by measuring the Z score using the ProSA-web server revealed that all the Z score values were significantly negative. The structural validation (allowed region and outlier) and quality score (Z score) are given in Table 1. The secondary structural details of the predicted proteins with their values are given in Table 2).

**Table 1:** Protein structure validation with quality Z score.

Sl no.	Gene ID	Gene	Ramachandran plot analysis (%)		Quality Z score
**1**	treatment_transcript_1371 betaine carnitine choline family	*opuCB*	Allowed region	98.8	-4.09
			Outlier	1.2	
**2**	treatment_transcript_6679 proline betaine transporter	*proP*	Allowed region	99.3	-5.33
			Outlier	0.7	
**3**	treatment_transcript_4687 betaine aldehyde dehydrogenase	*betB*	Allowed region	100	-1.08
			Outlier	0.0	
**4**	treatment_transcript_1069 bacterial abc transporter protein	*artI*	Allowed region	95.7	-6.20
			Outlier	4.3	
**5**	treatment_transcript_1432 dass family	*citT*	Allowed region	96.6	-6.12
			Outlier	3.4	
**6**	treatment_transcript_5134 ATP synthase gamma subunit	*atpE*	Allowed region	98.0	-3.80
			Outlier	2.0	
**7**	treatment_transcript_194 succinate dehydrogenase iron-sulfur subunit	*sdhB*	Allowed region	98.0	-3.80
			Outlier	2.0	
**8**	treatment_transcript_6954 succinate dehydrogenase flavoprotein subunit	*sdhA*	Allowed region	98.9	-4.05
			Outlier	1.1	
**9**	treatment_transcript_7764 succinate semialdehyde dehydrogenase	*gabD*	Allowed region	98.1	-2.27
			Outlier	1.9	
**10**	treatment_transcript_10031 fumarate class ii	*fumC*	Allowed region	100.0	-1.65
				0.0	

**Table 2:** Predicted structural details of salt tolerance related functional genes.

Sl no.	Gene ID	Gene name	Structural details and their values	
1	treatment_transcript_1371 betaine carnitine choline family	*opuCB*	No of Helices	17
			No of Strands	0
			No of Turn	16
			H-Bonds	227
2	treatment_transcript_6679 proline betaine transporter	*proP*	No of Helices	7
			No of Strands	0
			No of Turn	9
			H-Bonds	117
3	treatment_transcript_4687 betaine-aldehyde dehydrogenase	*betB*	No of Helices	5
			No of Strands	6
			No of Turn	9
			H-Bonds	92
4	treatment_transcript_1069 bacterial abc transporter protein	*artI*	No of Helices	3
			No of Strands	6
			No of Turn	13
			H-Bonds	44
5	treatment_transcript_1432 dass family	*citT*	No of Helices	2
			No of Strands	0
			No of Turn	7
			H-Bonds	29
6	treatment_transcript_5134 ATP synthase gamma subunit	*atpE*	No of Helices	2
			No of Strands	4
			No of Turn	12
			H-Bonds	35
7	treatment_transcript_194 succinate dehydrogenase iron-sulfur subunit	*sdhB*	No of Helices	9
			No of Strands	6
			No of Turn	21
			H-Bonds	91
8	treatment_transcript_6954 succinate dehydrogenase flavoprotein subunit	*sdhA*	No of Helices	12
			No of Strands	4
			No of Turn	22
			H-Bonds	120
9	treatment_transcript_7764 succinate-semialdehyde dehydrogenase	*gabD*	No of Helices	5
			No of Strands	7
			No of Turn	17
			H-Bonds	91
10	treatment_transcript_10031 fumarate class ii	*fumC*	No of Helices	7
			No of Strands	4
			No of Turn	13
			H-Bonds	128

## Discussion

In the present study, the SOLiD 4.0 sequencing technology was used to analyze the transcriptome of *Staphylococcus* sp. Altogether 11,796 and 10,973 transcripts were assembled. Large scale data generated in this study would place an excellent foundation for further research into the novel gene discovery, inheritances, genomics, as well as, proteomics of *Staphylococcus* sp. Likewise, several researchers generated massive data regarding stress tolerance of different genera which revealed several essential genes and pathways related to those genes in their respective metabolic pathways [55, 27, 56]. Furthermore, the transcriptome sequences acquired in this study provide the conscious of the genetic makeup of the bacteria and the sequences created here are convenient for additional research into salt tolerance.

The scarcity of comprehensive genome information to be used as reference guided us to perform transcriptome assembly from the short raw reads using several *de novo* assembly programs. Prior studies have revealed that multiple assemblies at altering k-mer values capture lower expressed transcripts after equating with a single k-mer assembly [57]. Earlier studies by Zerbino and Birney [42] have shown that a k-mer value as massive as this usually generates a more adjoining assembly of highly expressed RNAs.

Functional annotation of transcript contigs for both the samples was analyzed using BLASTx and GO mapping to understand the putative functions of each transcript at a whole-transcriptome level. The conclusion of the annotation presented maximum similarity with significant portions of the *Staphylococcus* sp. The BLASTx hits for each of the transcript contigs lie within the cut-off value 1e-06 having significant sequence similarity with protein nr databases at NCBI, whereas, a lower percentage of transcripts (12.85% of control and 12.4% of treated) remained un-annotated group in this study. Similar analyses were performed by Janz et al. [55] revealing the evolutionary adaptation of stress tolerance mechanisms by using pathway investigation of the transcriptome and metabolome of salt-sensitive as well as salt tolerant poplar species using GO analysis as a tool that agrees with the present work. In another study, Carvalho et al. [56] analyzed the ecologically relevant genes by performing the transcriptome *de novo* assembly from next-generation sequencing along with comparative analyses in a hexaploid salt marsh species through gene ontology studies using BLAST2GO tool.

The highest and the lowest CPB value for control and treated samples obtained in this study was found in tune with the reports analyzed by Hillier et al. [58] during the investigation of massively parallel sequencing of the *C*. *elegans* polyadenylated transcriptome.

The average GC content for control sample showed the comparatively low proportion of transcript contigs (29%) with GC content in the range of 35–40% while the treated sample showed a higher percentage of transcript contigs (31.28%) with GC content in the range of 35–40% which substantiate the reports of, Chen et al. [41].

The Kyoto encyclopedia of genes and genomes (KEGG) study with KEGG Automatic Annotation Server (KAAS) of the assembled transcripts shown putative members of metabolic pathways which may be involved in diverse metabolic pathways which is further involved in salt tolerance. Pathway enrichment using the KAAS server was able to find 10280 sequences associated with 212 KEGG pathways in control transcript contigs and 968 KEGG pathways related to 9612 sequences in treated transcript contigs. Moreover, it was also observed that transcripts from both the samples were involved in some common pathways responsible for salt tolerance including glycolysis/gluconeogenesis, oxidative phosphorylation, glutathione metabolism, etc. In a similar way, Tang et al. [59] studied the global gene expression of the seedlings of *Kosteletzkya virginia* responding to salt stress justifying the present study. The distribution of GO terms in the respective biological components *viz*. cellular component, biological process and molecular function enriched transcripts of both the sample. Comparing to the control transcripts, maximum transcripts from treated samples were associated to cellular process and metabolic process i.e. biological process, cell, and organelle i.e. cellular component and catalytic activity and binding i.e. a molecular function which tailored the increased actions of absorption, growth, and improvement in *Staphylococcus* sp. [60, 61]. Moreover, it was also observed that there was a significant fold increase) in the number of metabolic processes governed by each transcript of treated as compared to the control one. A remarkable finding about the transcripts related to metabolism which considerably enhanced (67.1%) is that, it specified metabolism as the main up-regulated function of the salt-tolerant *Staphylococcus* sp.

The KEGG pathway study showed that a variety of pathways was symbolized by the treated transcriptome data set. Various metabolic pathways including spliceosome, secondary metabolite biosynthesis, ribosome as well as ubiquitin-mediated proteolysis were the main symbolized pathways amongst the treated transcripts of *Staphylococcus* sp. The number and assortment of allocated GO categories and pathways afford excellent evidence of the enormous diversity of expressed transcripts tested from the salt-tolerant transcriptome of *Staphylococcus* sp. Salt treated transcripts of *Staphylococcus* sp. exhibited wide spectrum KEGG paths as compared to control one, signifying that these KEGG pathways may dynamically contribute to the salt stress. Moreover, previous studies have shown that the paths responsive to salt stress in many species could also upgrade the hostile things of salinity [62].

The genes characterized by transcriptome profiling may be accountable for the salt stress tolerance of the bacteria. The genes vary functionally as per the analysis based on the gene ontology study. Depending on the functional similarity, all the genes were classified in ten different groups which are more or less related to each other as they share some common pathways for the salt stress tolerance mechanism. Among them, the majority of the genes belong to the ABC transporter family. Other than that glycine betaine, symporter antiporter family, carrier protein family, dass family and glutamate family are some of them. A sum of 90 different genes has been found, which are functionally similar and upregulated due to the salt stress. The biological processes, cell components and molecular functions of all the ten groups have been documented from the gene ontology studies (S7 Table). The upregulated genes associated with salt-tolerant pathways (S8 Table) and the upregulated genes related to different metabolic pathways (S9 Table) have been documented. Some of the genes are common with prokaryotic as well as eukaryotic cellular pathways which show that the genes are to some extent similar to other higher groups of the living being concerning functionality. However, the stress mechanism is unique and almost all organisms (including plant, animal, etc.) respond practically similar to different types of abiotic stresses. Considering the fact, the study has an excellent impact on future studies of stress-responsive genes in bacteria. A sum of 1,288 and 1,133 transcript contigs was considered exclusively as novel transcript contigs in control and treated samples, respectively, as there are no reports in the current database regarding these transcript contigs. So, it can be assumed that no worker has characterized these salt transcripts to date. Some of the important genes of these transcripts might be involved in the stress tolerance mechanism. These studies can be established only after validating every transcript in the future, which is beyond the scope of this study.

In this study, most of the genes (viz. *abc3*, *mlaE*, *art1*, *nikB*, *opuBA*, *chiQ*) involved in the stress mechanism were found functionally associated with abc transporter family, i.e. membrane transport pathways of the bacteria. Some of the genes are associated with osmoprotectant *viz*. glycine betaine, sodium potassium transporters, trehalose, while rest of the genes are associated with dass family protein, abc atp-binding protein, abc permease protein, abc solute binding protein, abc substrate binding family 3, abc-2 type transporter, neurotransmitter family, amino acid carrier protein, etc. (S7 Table). In recent study, Xiong and co-workers [63] reported the genes responsible for membrane transport, abc transport, glycine betaine and proline in *E*. *coli* under salt stress which corroborates to our present study. A similar study has been performed by Choi et al. [27] on a strain of *Staphylococcus* sp. as the genus tolerates high NaCl concentration. A number of researchers have investigated glycine betaine gene, trehalose gene, ectoine gene, sodium, and hydrogen antiporters from different bacteria *viz*. *KatE*, *HPT* and *NPTII* from *Escherichia coli*[64–66], *BetS*from *Sinorhizobiummeliloti*[13],*ectABC*from *Chromohalobactersalexigens*[16, 67], *ectABC* from *Bacillus halodurans* [17], *codA* from *Arthrobacter globiformis* strain ATCC 8010 [68, 69], *a-aminoisobutyric acid (AIB)* from *Vibrio costicola* [70], *OpuC and OpuB* from *Listeria monocytogenes* [71], *ablA* from *Methanosarcinamazei*Go¨1 [72], *otsA/otsB*, *mpgS/mpgP* from *Thermus thermophilus* [73], *BetT* from *Pseudomonas syringae* [74], *proH*, *proJ and proA* from *Halobacillus halophilus* [75], *Mpgsmt-sdmt* from Halophilic Methanogen *Methanohalophilus portucalensis* [76], Bacterial mannitol 1-phosphate dehydrogenase (*mtlD*) gene frpm *mtlD* producing bacteria [77].

Based on the differential genes expression analysis, the upregulated and downregulated genes associated with salt stress were identified with more focus on upregulated salt stress tolerant genes. The genes consisted of both candidate salt stress tolerant genes as well as the genes associated with several metabolic pathways *viz*. tri-carboxylic acid cycle, transmembrane transport, carbohydrate transport, metal ion binding, oxidation-reduction, enzyme activity, signal transduction, response to antibiotics, nitrate assimilation and ATPase activity. Most of the salt stress tolerant genes were upregulated compared to the control group. Rest genes associated with several metabolic pathways got downregulated, which has been depicted in the heat map (Fig 6).In another study, Liu et al. [78] analyzed the transcriptome of *Shewanella oneidensis* MR-1 in response to elevated salt-stressed conditions and reported the genes that are involved in Na^+^ efflux and K^+^ accumulation, glutamate biosynthesis as well as respiration (tricarboxylic acid cycle) related genes were upregulated due to the stress in agreement with our present investigation.

Furthermore, gene ontology (GO) term annotation at protein level reproduced the quantity of GO terms, annotated for the treated transcripts corresponds to numerous cellular constituents, biological routes and molecular function are increased to quite a few folds in the control transcripts. This signifies that salt-tolerant bacteria survive high salt concentration by expression of a wide range of genes under which the bacteria can survive in such extreme conditions as evidenced by the presence of salt-tolerant transcripts. Apart from salt-tolerant transcripts, the presence of novel transcripts in the *Staphylococcus* sp. has opened up the door for further exploration of the putative functions of such candidates soon. There were few reports on ESTs from *Staphylococcus* sp. in NCBI, so this study would significantly contribute to enriching the inadequate genetic assets obtainable for reviewing *Staphylococcus* sp. Five salt stress-tolerant genes such as *opuCB*, *proP*, *betB*, *artI*, *citT* and five salt stress associated genes such as, *atpE*, *sdhB*, *sdhA*, *gabD*, *fumC* were analyzed. Homology modellling has been considered one of the most biologically significant approaches for protein structure prediction. In this study, the homology modelling approach was used to generate the 3D native structure of salt stress-tolerant/salt stress associated protein in *Staphylococcus* sp. (Figs 7 and 8). To predict the functionality of any protein, it is essential to identify its structure. In a separate study, Joshi et al. (2009) isolated and functionally validated high salinity stress tolerant genes from *Pisum sativum* along with the homology-based computational modelling of the proteins of the respective genes and the high degree of conservation with the conserved domains of their homologous partner which corroborates the present study [79]. Further, an in-depth structural analysis is required in our study to understand the significance of salt stress-tolerant functional genes which can lead to finding a novel way in genetic engineering.

The study identified some candidates and some novel salt-tolerant transcripts and successfully demonstrated the application of *de novo* assembly of a salt-tolerant microbe. It can be conjectured that effective transcriptional regulation is mostly accountable for salt tolerance in *Staphylococcus* sp. The result from the present study provides a valuable resource for future genetic diversity, an idea about salt adaptation studies and aid in the mining of some novel salt-tolerant genes. Besides, it will be very alluring to explore the exact function of genes concerned in the metabolic pathways improved during salt stress in *Staphylococcus* sp. Hence, it can be established that, in adjoining the future, the genetic engineering of alike candidate genes/pathways will make available a clear depiction of the reserve for the advancement of stress tolerance crop plants to meet global food security and sustainable food production in saline ecologies.

Several researchers reported numerous *Staphylococci* together with *S*. *saprophyticus*, *S*. *epidermidis* and other various *Staphylococcus* grow up in 10% NaCl conditions [80]. *S*. *piscifermentans*, *S*. *condimentii*, and *S*. *carnosus* can propagate at 15% NaCl [27] and *S*. *agnetis* can tolerate even upto 19% NaCl [39]. *Staphylococcus* sp. is predictable to experience altering ecological surroundings and stresses, including osmotic stress as *Staphylococcus* sp., exist in a variety of environments which include outside the natural environment, human host, food and animal. Consequently, resistance to osmotic stress would execute a significant part for the adaptation of *Staphylococcus* below high NaCl state [27]. Inagaki et al. [81] cited the studies of bacterial communities in geologic resources where they showed the malleability of microorganisms to a diversity of microhabitats and thus they consider microbes to be indigenous. Therefore, it is comprehensible that, the extent of bacterial diversity in the environment is still indefinite, which signifies the existence of many more functional bacterial components that might be recognized shortly from salt-tolerant microbes from marine as well as terrestrial ecosystems.

## Conclusion

The information attained in the present study may be used to categorize and clone the salt stress-tolerant genes and support in developing the salt stress-tolerant plant as well as fish varieties for augmenting the agricultural productivity, besides, to meet the demand of food in the near future.

## Supporting information

S1 Fig Annotated data distribution of *Staphylococcus* sp.transcript contigs for control and treated bacterial sample. (DOCX)

S2 Fig GO-level distribution (A) Control (B) Treated bacterial sample.
(DOCX)

S3 Fig Distribution of salt tolerant transcript contigs in control and treated sample. (DOCX)

S4 Fig Venn diagram showing the comparative analysis of salt tolerant transcript contigs in *Staphylococcus* sp.control and treated samples. (DOCX)

S5 Fig Distribution of novel transcript contigs in control and treated bacterial sample. (DOCX)

S6 Fig Gene ontology distribution (A) Control (B) Treated bacterial sample.
(DOCX)

S7 Fig GC content analysis of (A) Control (B) Treated transcript contigs.
(DOCX)

S1 Table Coverage per base statistics of *Staphylococcus* sp. control and treated samples. (DOCX)

S2 Table List of upregulated abc transporter genes unique to *Staphylococcus* sp. in response to salt stress. (DOCX)

S3 Table List of upregulated sodium antiporter genes unique to *Staphylococcus* sp. in response to salt stress. (DOCX)

S4 Table List of upregulated sodium symporter genes unique to *Staphylococcus* sp. in response to salt stress. (DOCX)

S5 Table List of upregulated glycine-betaine genes unique to *Staphylococcus* sp. in response to salt stress. (DOCX)

S6 Table List of upregulated trehalose genes unique to *Staphylococcus* sp. in response to salt stress. (DOCX)

S7 Table Classification of the gene ontology study of the functionally annotated genes. (DOCX)

S8 Table List of upregulated genes associated with salt tolerant pathways. (DOCX)

S9 Table List of upregulated genes associated with different metabolic pathways. (DOCX)

S10 Table SSR mining statistics in control and treated sample. (DOCX)

S11 Table Distribution of SSRs in different repeat types. (DOCX)

## References

[1] Pardo J.M., 2010 Biotechnology of water and salinity stress tolerance. Curr Opin Biotechnol 21, 185–196. 10.1016/j.copbio.2010.02.005 20189794

[2] Wang H., Miyazaki S., Kawai K., Deyholos M., Galbraith D.W., Bohnert H.J., 2003 Temporal progression of gene expression responses to salt shock in maize root. Plant Mol Biol 52, 873–891. 10.1023/a:1025029026375 13677474

[3] Nath IVA., Bharathi P.A.L., 2011 Diversity in transcripts and translational pattern of stress proteins in marine extremophiles. Extremophil 15, 129–153. 10.1007/s00792-010-0348-x 21210167

[4] Baati H., Amdouni R., Gharsallah N., 2010 Isolation and characterization of moderately halophilic bacteria from Tunisian solar saltern. Curr Microbiol 60, 157–161. 10.1007/s00284-009-9516-6 19826862

[5] Sleator R.D., Hill C., 2002 Bacterial osmoadaptation: the role of osmolytes in bacterial stress and virulence. FEMS Microbiol Rev 26, 49–71. 10.1111/j.1574-6976.2002.tb00598.x 12007642

[6] Sanjukta R.K., Farooqi M.S., Rai N., Rai A., Sharma N., Mishra D.C., et al 2013 Expression analysis of genes responsible for amino acid biosynthesis in halophilic bacterium *Salinibacter ruber*. Ind J Biochem Biophys 50, 177–185. 23898480

[7] Caton T.M., Witte L.R., Ngyuen H.D., Buchheim J.A., Buchheim M.A., Schneegurt M.A., 2004 Halotolerant aerobic heterotrophic bacteria from the great salt plains of Oklahoma. Microb Ecol 48, 449–462. 10.1007/s00248-004-0211-7 15696379

[8] Jiang H.C., Dong H.L., Zhang G.X., Yu B.S., Chapman L.R., Fields M.W., 2006 Microbial diversity in water and sediment of Lake Chaka, an Athalassohaline Lake in northwestern China. Appl Environ Microbiol 72, 3832–3845. 10.1128/AEM.02869-05 16751487 PMC1489620

[9] Tsiamis G., Katsaveli K., Ntougias S., Kyrpides N., Andersen G., Piceno Y., et al 2008 Prokaryotic community profiles at different operational stages of a Greek solar saltern. Res Microbiol 159, 609–627. 10.1016/j.resmic.2008.09.007 18976703

[10] Xiang W.L., Guo J.H., Feng W., Huang M., Chen H., Zhao J., et al 2008 Community of extremely halophilic bacteria in historic Dagong brine well in southwestern China.World J Microbiol Biotechnol 24, 2297–2305.

[11] Behera B.K., Das P., Maharana J., Meena D.K., Sahu T.K., Rao A.R., et al 2014 Functional screening and molecular characterization of halophilic and halotolerant bacteria by 16S rRNA gene sequence analysis. Proc Natl Acad Sci India Sect B Biol Sci 85, 957–964.

[12] Hagemann M., Schoor A., Jeanjean R., Zuther E., Joset F., 1997 The stpA gene from *Synechocystis* sp. strain PCC 6803 encodes the glucosylglycerol-phosphate phosphatase involved in cyanobacterial osmotic response to salt shock. J Bacteriol. 179, 1727–1733. 10.1128/jb.179.5.1727-1733.1997 9045835 PMC178888

[13] Boscari A., Mandon K., Dupont L., Poggi M.C., Rudulier D.L., 2002 BetS Is a major glycine betaine/proline betaine transporter required for early osmotic adjustment in *Sinorhizobium meliloti*. J Bacteriol 184, 2654–2663. 10.1128/JB.184.10.2654-2663.2002 11976294 PMC135037

[14] Purvis J.E., Yomano L.P., Ingram L.O. 2005 Enhanced trehalose production improves growth of *Escherichia coli* under osmotic stress. Appl Environ Microbiol 71, 3761–3769. 10.1128/AEM.71.7.3761-3769.2005 16000787 PMC1168978

[15] Turan S., Cornish K., Kumar S., 2012 Salinity tolerance in plants: Breeding and genetic engineering. Aus J Crop Sci 6, 1337–1348.

[16] Schubert T., Maskow T., Benndorf D., Harms H., Breuer U., 2007 Continuous synthesis and excretion of the compatible solute ectoine by a transgenic, nonhalophilic bacterium. App Env Microbiol 73, 3343–3347. 10.1128/AEM.02482-06 PMC1907108 17369334

[17] Rajan L.A., Joseph T.C., Thampuran N., James R., Ashok Kumar K., Viswanathan C., et al 2008 Cloning and heterologous expression of ectoine biosynthesis genes from *Bacillus halodurans* in *Escherichia coli*. Biotechnol Lett. 30, 1403–1407. 10.1007/s10529-008-9688-3 18488150

[18] Ofer N., Wishkautzan M., Meijler M., Wang Y., Speer A., Niederweis M., et al 2012 Ectoine biosynthesis in *Mycobacterium smegmati*. App Env Microbiol 78, 7483–7486. 10.1128/AEM.01318-12 PMC3457100 22885758

[19] Li P., Ponnala L., Gandotra N., Wang L., Si Y., Tausta S.L., et al 2010 The developmental dynamics of the maize leaf transcriptome. Nature Genet 42, 1060–1067. 10.1038/ng.703 21037569

[20] Shi C.Y., Yang H., Wei C.L., Yu O., Zhang Z.Z., Jiang C.J., et al 2011 Deep sequencing of the *Camellia sinensis* transcriptome revealed candidate genes for major metabolic pathways of tea-specific compounds. BMC Genom 12, 131–149. 10.1186/1471-2164-12-131 PMC3056800 21356090

[21] Wang Z., Gerstein M., Snyder M., 2009 RNA-Seq: a revolutionary tool for transcriptomics. Nat Rev Gen 10, 57–63. 10.1038/nrg2484 PMC2949280 19015660

[22] Croucher N.J., Thomson N.R., 2010 Studying bacterial transcriptomes using RNA-seq. Curr Opin Microbiol 13, 619–624. 10.1016/j.mib.2010.09.009 20888288 PMC3025319

[23] Sharma C.M., Vogel J., 2014 Differential RNA-seq: the approach behind and the biological insight gained. Cur Opin Microbiol 19, 97–105. 10.1016/j.mib.2014.06.010 25024085

[24] Das B.K., Chakraborty H.J., Rout A.K., Behera B.K., 2019 De novo whole transcriptome profiling of *Edwardsiella tarda* isolated from infected fish (*Labeo catla*). Gene 701, 152–160. 10.1016/j.gene.2019.03.028 30910556

[25] Lorenzo L. de., Merchan F., Blanchet S., Megı´as M., Frugier F., Crespi M., et al 2007 Differential expression of the TFIIIA regulatory pathway in response to salt stress between Medicago truncatula genotypes. Plant Physiol 145, 1521–1532. 10.1104/pp.107.106146 17951460 PMC2151693

[26] Yao D., Zhang X., Zhao X., Liu C., Wang C., Zhang Z., et al 2011 Transcriptome analysis reveals salt-stress-regulated biological processes and key pathways in roots of cotton (*Gossypium hirsutum* L.). Genom 98, 47–55. 10.1016/j.ygeno.2011.04.007 21569837

[27] Choi S., Jung J., Jeon C.O., Park W., 2014 Comparative genomic and transcriptomic analyses of NaCl-tolerant *Staphylococcus* sp. OJ82 isolated from fermented seafood. Appl Microbiol Biotechnol 98, 807–822. 10.1007/s00253-013-5436-2 24346282

[28] Liu X., Luo Y., Mohamed O.A., Liu D., Wei G., 2014 Global transcriptome analysis of *Mesorhizobium alhagi* CCNWXJ12-2 under salt stress. BMC Microbiol 14, 319. 10.1186/s12866-014-0319-y PMC4302635 25539655

[29] Solheim M., La Rosa S.L., Mathisen T., Snipen L.G., Nes I.F., Brede D.A., 2014 Transcriptomic and functional analysis of NaCl-induced stress in *Enterococcus faecalis*. PLoS One 9, e94571 10.1371/journal.pone.0094571 24755907 PMC3995695

[30] Miao Z., Xu W., Li D., Hu X., Liu J., Zhang R., et al 2015 *De novo* transcriptome analysis of *Medicago falcata* reveals novel insights about the mechanisms underlying abiotic stress-responsive pathway. BMC Genom 16, 818. 10.1186/s12864-015-2019-x PMC4615886 26481731

[31] Razzauti M., Galan M., Bernard M., Maman S., Klopp C., Charbonnel N., et al 2015 A comparison between transcriptome sequencing and 16S metagenomics for detection of bacterial pathogens in wildlife. PLoSNegl Trop Dis 9, e0003929. 10.1371/journal.pntd.0003929 PMC4540314 26284930

[32] Madigan M, Martinko J, *eds* (2005). *Brock Biology of Microorganisms (11th ed.)*. Prentice Hall.

[33] Lambert L.H., Cox T., Mitchell K., Rosselló-Mora R.A., Del Cueto C., Dodge D.E., et al 1998 *Staphylococcus succinus* sp. nov., isolated from Dominican amber. Int J Syst Evol Microbiol 48, 511–518. 10.1099/00207713-48-2-511 9731292

[34] Hajek V., Ludwig W., Schleifer K.H., Springer N., Zitzelsberger W., Kroppenstedt R.M., et al 1992 *Staphylococcus muscae*, a new species isolated from flies. Int J Syst Evol Microbiol 42, 97–101. 10.1099/00207713-42-1-97 1371067

[35] Gunn B.A., Colwell R.R., 1983 Numerical taxonomy of *Staphylococci* isolated from the marine environment. Int J Syst Evol Microbiol 33, 751–759.

[36] Tanasupawat S., Hashimoto Y., Ezaki T., Kozaki M., Komagata K., 1992 *Staphylococcus piscifermentans* sp. nov., from fermented fish in Thailand. Int J Syst Evol Microbiol 42, 577–581. 10.1099/00207713-42-4-577 1390108

[37] Vernozy-Rozand C., Mazuy C., Meugnier H., Bes M., Lasne Y., Fiedler F., et al 2000 *Staphylococcus fleurettii* sp. nov., isolated from goat's milk cheeses. Int J Syst Evol Microbiol 50, 1521–1527. 10.1099/00207713-50-4-1521 10939659

[38] Tsai M., Ohniwa R.L., Kato Y., 2011 *Staphylococcus aureus* requires cardiolipin for survival under conditions of high salinity. BMC Microbiol 11, 13 10.1186/1471-2180-11-13 21241511 PMC3030509

[39] Taponen S., Supre K., Piessens V., Van Coillie E., De Vliegher S., Koort J.M., 2012 *Staphylococcus agnetis* sp. nov., a coagulase-variable species from bovine subclinical and mild clinical mastitis. Int J Syst Evol Microbiol 62, 61–65. 10.1099/ijs.0.028365-0 21335502

[40] Ming-xiang Z., Rong-rong Z., Wen-jun W., Ning-jie Z., Wen-en L., Fu-ping H., et al 2012 Antimicrobial resistance and molecular epidemiological characteristics of clinical isolates of *Staphylococcus aureus* in Changsha area. Chin Med J.125, 2289–2294. 22882850

[41] Chen S., Zhou R., Huang Y., Zhang M., Yang G., Zhong C., et al 2011 Transcriptome sequencing of a highly salt tolerant mangrove species *Sonneratia alba* using Illumina platform. Marine Genom 4,129–136. 10.1016/j.margen.2011.03.005 21620334

[42] Zerbino D.R., Birney E., 2008 Velvet: Algorithms for *de novo* short read assembly using de Bruijn graphs. Genome Res 18, 821–829. 10.1101/gr.074492.107 18349386 PMC2336801

[43] Li W., Godzik A., 2006 Cd-hit: a fast program for clustering and comparing large sets of protein or nucleotide sequences. Bioinfo 22, 1658–1659. 10.1093/bioinformatics/btl158 16731699

[44] Conesa A., Götz S., García-Gómez J.M., Terol J., Talónand M., Robles M., 2005 Blast2GO: a universal tool for annotation, visualization and analysis in functional genomics research. Bioinfo 21, 3674–3676. 10.1093/bioinformatics/bti610 16081474

[45] Huntley P.R., Binns D., Dimmer E., Barell D., O'Donovan C., Apweiler R., 2009 QuickGO: a user tutorial for the web-based gene ontology browser. European Bioinformatics Institute, Wellcome Trust Genome Campus, Hinxton, Cambridge, UK. 10.1093/database/bap010 PMC2794795 20157483

[46] Hunter S., Jones P., Mitchell A., Apweiler R., Attwood K.T., Bateman A. 2012 InterPro in 2011 New developments in the family and domain prediction database. Nucleic Acid Res 40, 306–312. 10.1093/nar/gkr948 PMC3245097 22096229

[47] Kanehisa M., 1997 A database for post-genome analysis. Trends Genet 13, 375–376. 10.1016/s0168-9525(97)01223-7 9287494

[48] Moriya Y., Itoh M., Okuda S., Yoshizawa A., Kanehisa M., 2007 KAAS: an automatic genome annotation and pathway reconstruction server. Nucleic Acids Res 35, 182–185. 10.1093/nar/gkm321 PMC1933193 17526522

[49] Prasath D., Karthika R., Habeeba N.T., Suraby E.J., Rosana O.B., Shaji A., et al 2014 Comparison of the transcriptomes of ginger (*Zingiber officinaleRosc*.) and mango ginger (*Curcuma amadaRoxb*.) in response to the bacterial wilt infection. Plos One 9, e99731 10.1371/journal.pone.0099731 24940878 PMC4062433

[50] Sali A., Potterton L., Yuan F., van Vlijmen H., Karplus M., 1995 Evaluation of comparative protein modelling by MODELLER. Proteins 23, 318–326. 10.1002/prot.340230306 8710825

[51] Laskowski R.A., MacArthur M.W., Moss D.S., Thornton J.M., 1993 PROCHECK: A program to check the stereochemical quality of protein structures. J Appl Cryst 26, 283–291.

[52] Sippl M.J., 1993 Recognition of errors in three-dimensional structures of proteins. Proteins 17, 355–362. 10.1002/prot.340170404 8108378

[53] Wiederstein M., Sippl M.J., 2007 ProSA-web: interactive web service for the recognition of errors in three-dimensional structures of proteins. Nucl Acids Res 35, 407–410. 10.1093/nar/gkm290 PMC1933241 17517781

[54] Sayle R., Milner-White E.J., 1995 "RasMol: Biomolecular graphics for all". Trend Biochem Sci 20, 374 10.1016/s0968-0004(00)89080-5 7482707

[55] Janz D., Behnke K., Schnitzler P.J., Kanawati B., Schmitt-Kopplin P., Polle A., 2010 Pathway analysis of the transcriptome and metabolome of salt sensitive and tolerant poplar species reveals evolutionary adaption of stress tolerance mechanisms. BMC Plant Biol 10, 150 10.1186/1471-2229-10-150 20637123 PMC3095294

[56] Carvalho J.F., de, Poulain J, Silva C. Da., Wincker P., Michon-Coudouel S., Dheilly A., et al 2013 Transcriptome *de novo* assembly from next-generation sequencing and comparative analyses in the hexaploid salt marsh species *Spartina maritima* and *Spartina alterniflora* (Poaceae). Heredity 110, 181–193. 10.1038/hdy.2012.76 23149455 PMC3554450

[57] Surget-Groba Y., Montoya-Burgos J.I., 2010 Optimization of *de novo* transcriptome assembly from next-generation sequencing data. Genome Res 20, 1432–1440. 10.1101/gr.103846.109 20693479 PMC2945192

[58] Hillier L.D.W., Reinke V., Green P., Hirst M., Marra M.A., Waterston R.H., 2009 Massively parallel sequencing of the polyadenylated transcriptome of *C*. *elegans*. Genom Res 19, 657–666. 10.1101/gr.088112.108 PMC2665784 19181841

[59] Tang X., Wang H., Shao C., Shao H., 2015 Global gene expression of *Kosteletzkya virginica* seedlings responding to salt stress. PLoS One 10, e0124421 10.1371/journal.pone.0124421 25901608 PMC4406580

[60] Huan P., Wang H., Liu B., 2011 Transcriptomic analysis of the clam *Meretrix meretrix* on different larval stages. Marine Biotechnol 14, 1–10. 10.1007/s10126-011-9389-0 21603879

[61] Zhu J.Y., Zhao N., Yang B., 2012 Global transcriptome profiling of the pine shoot beetle, *Tomicus yunnanensis* (Coleoptera: Scolytinae). PLoS One 7, e32291 10.1371/journal.pone.0032291 22384206 PMC3285671

[62] Basyuni M., Baba S., Inafuku M., Iwasaki H., Kinjo K., Oku H., 2009 Expression of terpenoid synthase mRNA terpenoid content in salt stressed mangrove. J Plant Physiol 166, 1786–1800. 10.1016/j.jplph.2009.05.008 19535167

[63] Xiong W., Luo W., Zhang X., Pan X., Zengd X., Yao C., et al 2019 High expression of toxic *Streptomyces* phospholipase D in *Escherichia coli* under salt stress and its mechanism. Biiomol Eng Bioeng Biochem Biofuel Food 10.1002/aic.16856

[64] Prodhan S.H., Hossain A., Nagamiya K., Komamine A., Morishima H., 2008 Improved salt tolerance and morphological variation in indica rice (*Oryza sativa* L) transformed with a catalase gene from *E coli*. Plant Tissue Cult Biotech 18, 57–63.

[65] Joseph T.C., Rajan L.A., Thampuran N., James R., 2010 Functional characterization of trehalose biosynthesis genes from *E coli*: An osmolyte involved in stress tolerance. Mol Biotechnol 46, 20–25. 10.1007/s12033-010-9259-4 20217281

[66] Padilla L., Morbach S., Kramer R., Agosin E., 2004 Impact of heterologous expression of *Escherichia coli* UDP-glucose pyrophosphorylase on trehalose and glycogen synthesis in *Corynebacterium glutamicum*. App Env Microbiol 70, 3845–3854. 10.1128/AEM.70.7.3845-3854.2004 PMC444832 15240254

[67] Calderon M.I., Vargas C., Rojo F., Iglesias-Guerra F., Csonka L.N., Ventosa A., et al 2004 Complex regulation of the synthesis of the compatible solute ectoine in the halophilic bacterium *Chromohalobacter salexigens* DSM 3043. Microbiol 150, 3051–3063. 10.1099/mic.0.27122-0 15347763

[68] Fan F., Ghanem M., Gadda G., 2004 Cloning, sequence analysis, and purification of choline oxidase from *Arthrobacter globiformis*: a bacterial enzyme involved in osmotic stress tolerance. Arch Biochem Biophys 42, 149–158. 10.1016/j.abb.2003.10.003 14678796

[69] Goel D., Singh A.K., Yadav V., Babbar S.B., Murata N., Bansal K.C., 2011 Transformation of tomato with a bacterial coda gene enhances tolerance to saltand water stresses. J Plant Physiol 168, 1286–1294. 10.1016/j.jplph.2011.01.010 21342716

[70] Kushner D.J., Hamaide F., MacLeod R.A., 1983 Development of salt resistant active transport in a moderately halophilic bacterium. J Bacteriol 153, 1163–1171. 6826519 10.1128/jb.153.3.1163-1171.1983 PMC221759

[71] Fraser K.R., Harvie D., Coote P.J., O’Byrne C.P., 2000 Identification and characterization of an ATP binding cassette L-carnitine transporter in *Listeria monocytogenes*. App Env Microbiol 66, 4696–4704. 10.1128/aem.66.11.4696-4704.2000 PMC92368 11055912

[72] Pfluger K., Baumann S., Gottschalk G., Lin W., Santos H., Muller V., 2003 Lysine-2, 3- aminomutase and -lysine acetyltransferase genes of methanogenic archaea are salt induced and are essential for the biosynthesis of *N–*acetyl- lysine and growth at high salinity. App Env Microbiol 69, 6047–6055. 10.1128/AEM.69.10.6047-6055.2003 PMC201229 14532061

[73] Alarico S., Empadinhas N., Simões C., Silva Z., Henne A., Mingote A., et al 2005 Distribution of genes for synthesis of trehalose and mannosylglycerate in *Thermus* spp and direct correlation of these genes with halotolerance. Appl Environ Microbiol 71, 2460–2466. 10.1128/AEM.71.5.2460-2466.2005 15870334 PMC1087547

[74] Chen C., Beattie G.A., 2008 *Pseudomonas syringae*BetT is a low-affinity choline transporter that is responsible for superior osmoprotection by choline over glycine betaine. J Bacteriol 190, 2717–2725. 10.1128/JB.01585-07 18156257 PMC2293270

[75] Saum S.H., Muller V., 2007 Salinity dependent switching of osmolyte strategies in a moderately halophilic bacterium: glutamate induces proline biosynthesis in *Halobacillus halophilus*. J Bacteriol 189, 6968–6975. 10.1128/JB.00775-07 17660292 PMC2045198

[76] Lai S.J., Lai M.C., 2011 Characterization and regulation of the osmolyte betaine synthesizing enzymes GSMT and SDMT from halophilic methanogen *Methanohalophilus portucalensis*. PLoS ONE 6, e25090 10.1371/journal.pone.0025090 21949863 PMC3176816

[77] Rahnama H., Vakilian H., Fahimi H., Ghareyazie B., 2011 Enhanced salt stress tolerance in transgenic potato plants (*Solanum tuberosum* L) expressing a bacterial mtlD gene. Acta Physiol Plant 33, 1521–1532.

[78] Liu Y., Gao W., Wang Y., Wu L., Liu X., Yan T., et al 2005 Transcriptome analysis of *Shewanella oneidensis* MR-1 in response to elevated salt conditions. J Bact 187, 2501–2507. 10.1128/JB.187.7.2501-2507.2005 15774893 PMC1065217

[79] Joshi A., Dang H.Q., Vaid N., Tuteja N., 2009 Isolation of high salinity stress tolerant genes from *Pisum sativum* by random overexpression in *Escherichia coli* and their functional validation. Plant Signal Behav 4, 400–412. 10.4161/psb.4.5.8387 19816097 PMC2676750

[80] Schleifer K.H., Kloos W.E., 1975 Isolation and characterization of *staphylococci* from human skin I Amended description of *Staphylococcus epidermidis* and *Staphylococcus saprophyticus* and descriptions of three new species: *Staphylococcus cohnii*, *Staphylococcus haemolyticus*, and *Staphylococcus xylous*. Int J Syst Evol Microbiol 25, 50f–61.

[81] Inagaki F., Suzuki M., Takai K., Oida H., Sakamoto T., Aoki K., et al 2003 Microbial Communities Associated with Geological Horizons in Coastal Subseafloor Sediments from the Sea of Okhotsk. Appl Environ Microbiol 69, 7224–7235. 10.1128/AEM.69.12.7224-7235.2003 14660370 PMC309994

