# Supplementary material for: De novo transcriptome analysis of halotolerant bacterium Staphylococcus sp. strain P-TSB-70 isolated from East coast of India: In search of salt stress tolerant genes
Source: PLoS One. 2020 Feb 10;15(2):e0228199. doi: 10.1371/journal.pone.0228199 (PMC7010390; doi:10.1371/journal.pone.0228199)
Supplement: S1 Fig — (DOCX) [file pone.0228199.s001.docx]

**
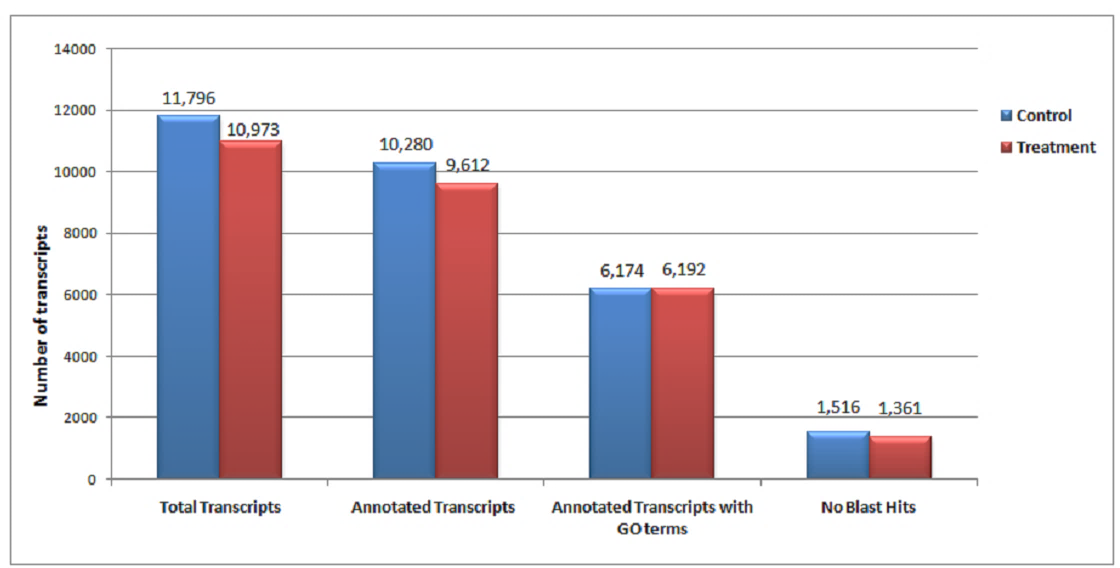
**

**S1 Fig. Annotated data distribution of *Staphylococcus* sp. transcript contigs for control and treated bacterial sample**
