# Supplementary figures and images for: De novo transcriptome analysis of halotolerant bacterium Staphylococcus sp. strain P-TSB-70 isolated from East coast of India: In search of salt stress tolerant genes
Source: PLoS One. 2020 Feb 10;15(2):e0228199. doi: 10.1371/journal.pone.0228199 (PMC7010390; doi:10.1371/journal.pone.0228199)

**
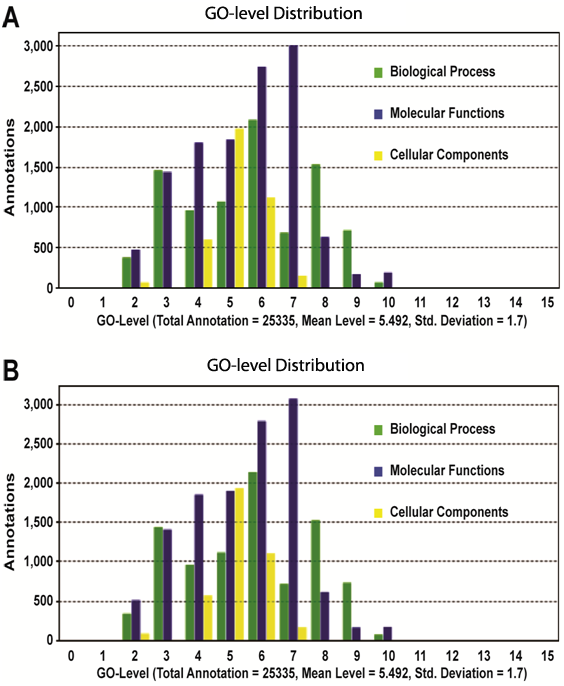
**

**S2 Fig. GO-level distribution (A) Control (B) Treated bacterial sample**

Supplement: S2 Fig — GO-level distribution (A) Control (B) Treated bacterial sample. (DOCX) [file pone.0228199.s002.docx]

**
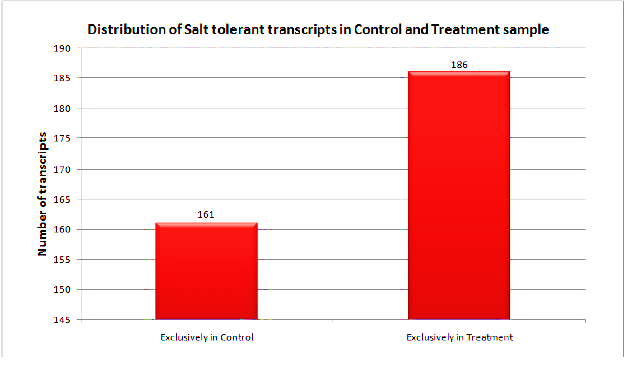
**

**S3 Fig.**  **Distribution of salt tolerant transcript contigs in Control and Treated sample**

Supplement: S3 Fig — (DOCX) [file pone.0228199.s003.docx]

**
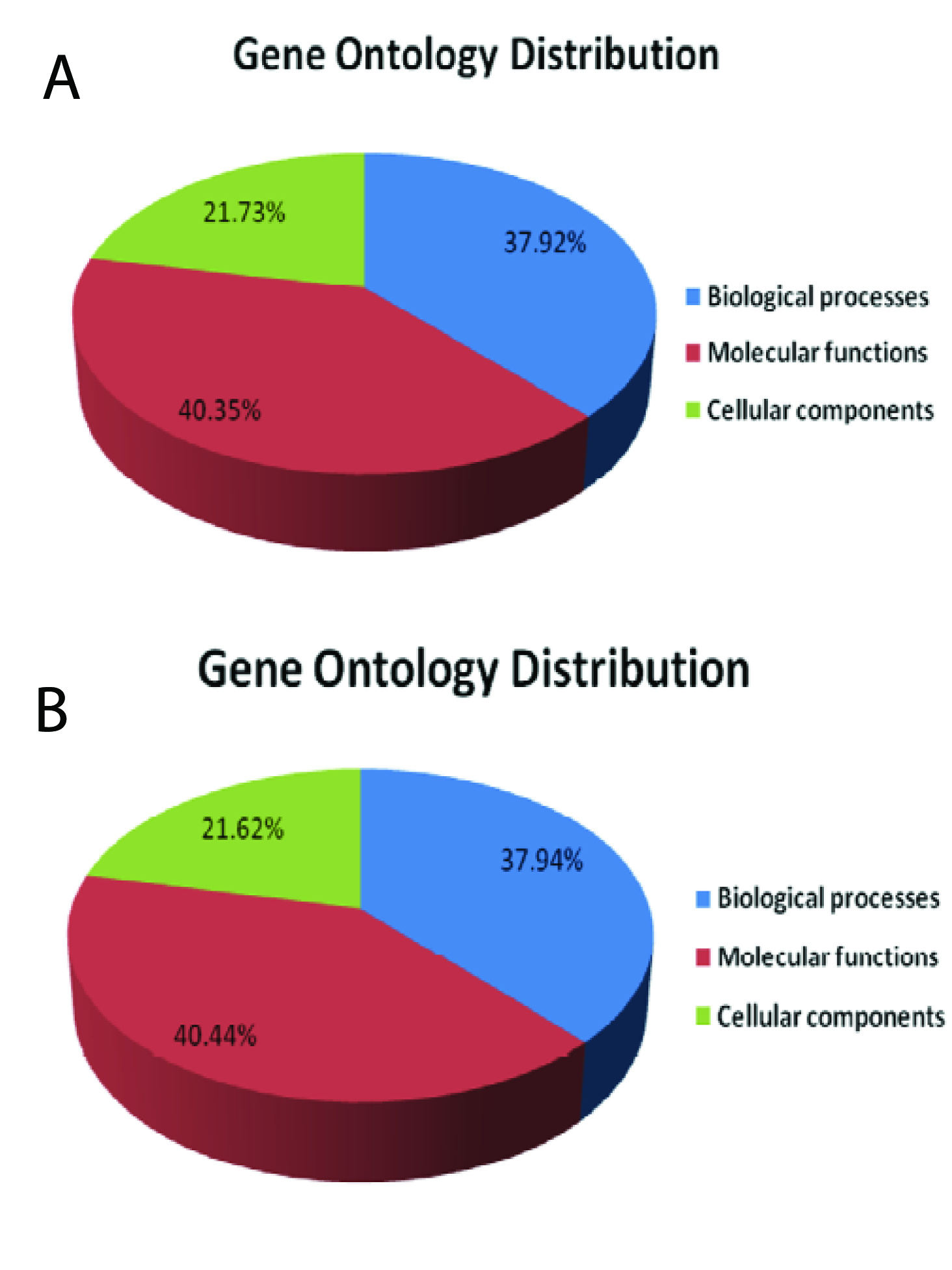
**

**S6 Fig. Gene ontology distribution (A) Control (B) Treated bacterial sample**

Supplement: S6 Fig — Gene ontology distribution (A) Control (B) Treated bacterial sample. (DOCX) [file pone.0228199.s006.docx]

**
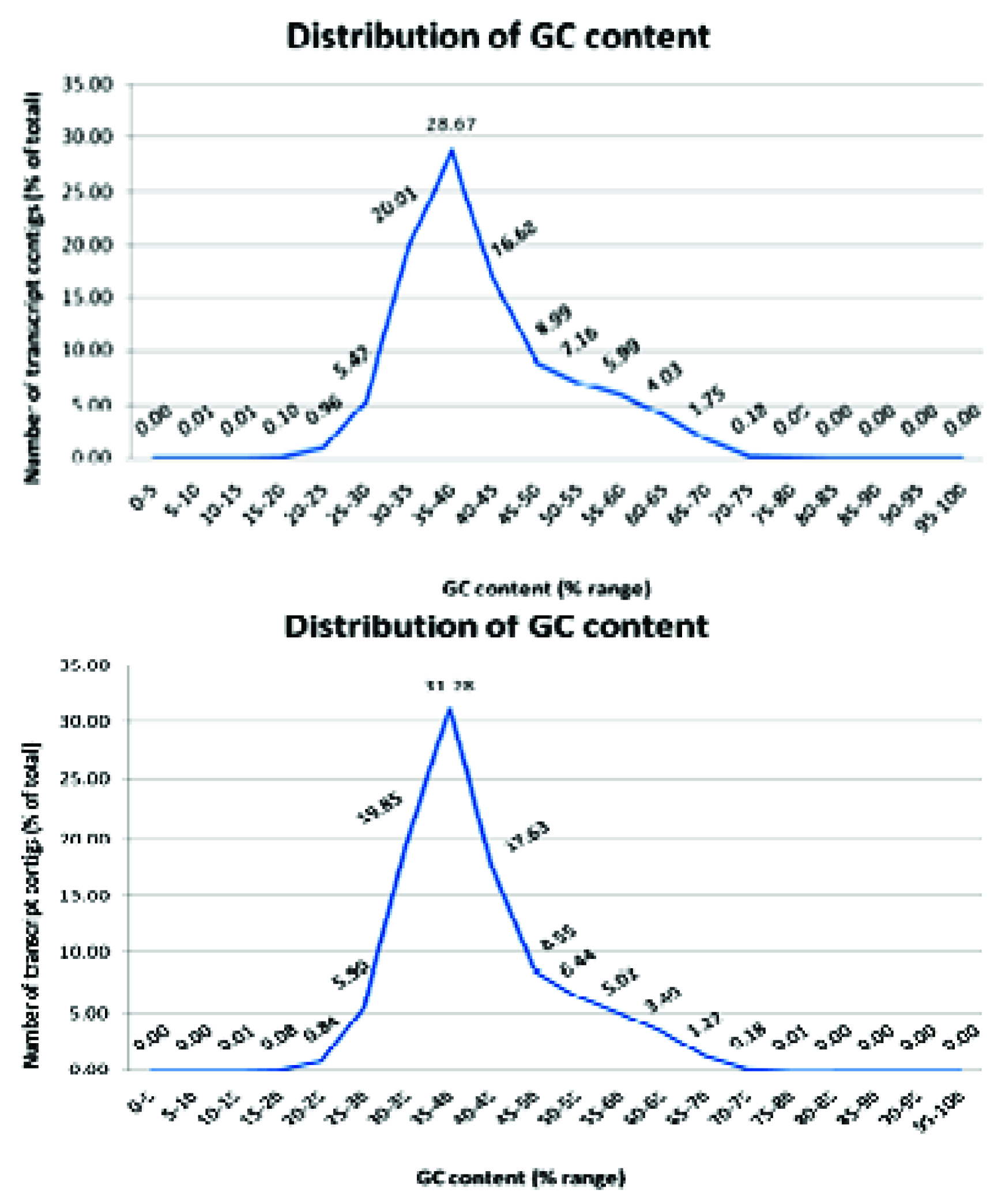
**

**S7 Fig.**  **GC content analysis of (A) Control (B) Treated transcript contigs**

Supplement: S7 Fig — GC content analysis of (A) Control (B) Treated transcript contigs. (DOCX) [file pone.0228199.s007.docx]
