# Supplementary material for: De novo transcriptome analysis of halotolerant bacterium Staphylococcus sp. strain P-TSB-70 isolated from East coast of India: In search of salt stress tolerant genes
Source: PLoS One. 2020 Feb 10;15(2):e0228199. doi: 10.1371/journal.pone.0228199 (PMC7010390; doi:10.1371/journal.pone.0228199)
Supplement: S4 Fig — (DOCX) [file pone.0228199.s004.docx]

**S4 Fig. Venn diagram showing the comparative analysis of salt tolerant transcript contigs in *Staphylococcus* sp.control**

**and treated samples**
