# Supplementary material for: De novo transcriptome analysis of halotolerant bacterium Staphylococcus sp. strain P-TSB-70 isolated from East coast of India: In search of salt stress tolerant genes
Source: PLoS One. 2020 Feb 10;15(2):e0228199. doi: 10.1371/journal.pone.0228199 (PMC7010390; doi:10.1371/journal.pone.0228199)
Supplement: S1 Table — (DOCX) [file pone.0228199.s008.docx]

**S1 Table. Coverage per base statistics of *Staphylococcus* sp. control and treated samples**

| **Description** | **Control** | **Treated** |
| --- | --- | --- |
| Highest Range of CPB | 39804.42 | 34714.94 |
| Lowest Range of CPB | 0.007582 | 0.002017 |
