# Supplementary material for: De novo transcriptome analysis of halotolerant bacterium Staphylococcus sp. strain P-TSB-70 isolated from East coast of India: In search of salt stress tolerant genes
Source: PLoS One. 2020 Feb 10;15(2):e0228199. doi: 10.1371/journal.pone.0228199 (PMC7010390; doi:10.1371/journal.pone.0228199)
Supplement: S2 Table — (DOCX) [file pone.0228199.s009.docx]

**S2 Table. List of upregulated abc transporter genes unique to *Staphylococcus* sp. in response to salt stress**

| **Sl. no.** | **Gene ID** | **Functional annotation** | **Gene** | **Sequence length** | **Hit accession** | **E-Value** | **Similarity** | **Score** | **Alignment Length** | **Positives** | **Sequence Similar to Functional Target Genes** |
| --- | --- | --- | --- | --- | --- | --- | --- | --- | --- | --- | --- |
| 1 | gi\|319400891\|gb\|EFV89110.1\|hydrophobic membrane protein ZurM | abc 3 transport family protein | *abc3* | 125 | EFV89110 | 6.96E-021 | 100 | 78.95 | 41 | 41 | 2 |
| 2 | gi\|365232221\|gb\|EHM73228.1\|ABC transporter transmembrane region | abcatp-binding protein | *Rv1747* | 478 | EHM73228 | 1.83E-095 | 99 | 284.26 | 148 | 147 | 95 |
| 3 | gi\|57866080\|ref\|YP_187695.1\|ABC transporter permease | abcpermease protein | *abc13p* | 340 | YP187695 | 8.16E-056 | 100 | 170.63 | 89 | 89 | 15 |
| 4 | gi\|57866202\|ref\|YP_187842.1\|ABC transporter substrate-binding protein | abc quaternary amine uptake transporter substrate-binding protein | *HMPREF1110_0377* | 334 | YP187842 | 4.11E-051 | 100 | 158.69 | 89 | 89 | 8 |
| 5 | gi\|365231674\|gb\|EHM72701.1\|ABC transporter, solute-binding protein | abc solute-binding protein | *TCCBUS3UF1_15170* | 278 | EHM72701 | 8.34E-045 | 100 | 145.21 | 69 | 69 | 2 |
| 6 | gi\|365231263\|gb\|EHM72319.1\|ABC transporter, substrate-binding protein, family 3 | abc substrate-binding family 3 | *ECBG_00399* | 185 | EHM72319 | 6.71E-034 | 89 | 110.92 | 66 | 59 | 3 |
| 7 | gi\|251796365\|ref\|YP_003011096.1\|ABC transporter | abc transporter | *yojI* | 155 | YP003011096 | 8.60E-012 | 100 | 53.53 | 25 | 25 | 7 |
| 8 | gi\|289661772\|ref\|ZP_06483353.1\|ABC transporter ATP-binding protein | abc transporter atp-binding protein | *yojI* | 249 | ZP06483353 | 2.98E-005 | 44 | 37.35 | 56 | 25 | 4 |
| 9 | gi\|363420540\|ref\|ZP_09308632.1\|ABC transporter ATPase | abc transporter atpase | *abc transporter* | 106 | ZP09308632 | 0 | 65 | 30.42 | 29 | 19 | 1 |
| 10 | gi\|251809720\|ref\|ZP_04824193.1\|ABC superfamily ATP binding cassette transporter, ABC protein | abc transporter family protein | *mlaE* | 286 | ZP04824193 | 8.47E-058 | 100 | 181.8 | 88 | 88 | 1 |
| 11 | gi\|374320982\|ref\|YP_005074111.1\|unnamed protein product | abc transporter like protein | *VMR1* | 100 | AET57888 | 0 | 56 | 29.26 | 32 | 18 | 1 |
| 12 | gi\|345021646\|ref\|ZP_08785259.1\|ABC transporter permease | abc transporter permease | *Rv1747* | 125 | ZP08785259 | 0 | 69 | 30.03 | 33 | 23 | 1 |
| 13 | gi\|57866884\|ref\|YP_188561.1\|hypothetical protein SERP0983 | abc transporter permease protein | *Rv1747* | 189 | YP188561 | 1.70E-025 | 81 | 87.43 | 60 | 49 | 3 |
| 14 | gi\|288918620\|ref\|ZP_06412969.1\|ABC transporter related protein | abc transporter related protein | *gsiA_1* | 155 | ZP06412969 | 1.03E-006 | 56 | 33.88 | 46 | 26 | 2 |
| 15 | gi\|114327930\|ref\|YP_745087.1\|unnamed protein product | abc transporter substrate-binding protein | *yesO_1* | 121 | YP745087 | 0.01 | 61 | 29.65 | 26 | 16 | 1 |
| 16 | gi\|374395053\|gb\|EHQ66327.1\|ABC transporter transmembrane region | abc transporter transmembrane region | *CFIO01_03315* | 105 | EHQ66327 | 2.68E-010 | 100 | 50.83 | 25 | 25 | 1 |
| 17 | gi\|27467330\|ref\|NP_763967.1\|teichoic acid biosynthesis protein | abc-2 type transporter | *GLRG_09770* | 213 | NP763967 | 1.37E-023 | 98 | 85.89 | 61 | 60 | 1 |
| 18 | gi\|261407097\|ref\|YP_003243338.1\|ABC transporter-like protein | aerobactin biosynthesis family | *HMPREF0798_01551* | 128 | YP003243338 | 8.20E-006 | 67 | 35.04 | 37 | 25 | 1 |
| 19 | gi\|239989589\|ref\|ZP_04710253.1\|putative ABC transporter ATPase and permease component | aldoketoreductase | *yvgN_2* | 132 | ZP04710253 | 2.53E-004 | 65 | 32.73 | 26 | 17 | 1 |
| 20 | gi\|313632859\|gb\|EFR99809.1\|amino acid ABC transporter, permease protein | amino acid abcpermease protein | *yecS_3* | 206 | EFR99809 | 9.59E-004 | 64 | 31.96 | 42 | 27 | 1 |
| 21 | gi\|361056326\|gb\|AEV95130.1\|Amino acid ABC transporter, permease/substrate-binding protein | amino acid abcpermease substrate-binding protein | *gltJ* | 115 | AEV95130 | 0 | 65 | 29.65 | 35 | 23 | 1 |
| 22 | gi\|372270247\|ref\|ZP_09506295.1\|putative ATP-binding component of ABC transporter | atp-binding component of abc transporter | *abc2* | 110 | ZP09506295 | 0.01 | 62 | 29.26 | 32 | 20 | 1 |
| 23 | gi\|242243057\|ref\|ZP_04797502.1\|ABC superfamily ATP binding cassette transporter, membrane protein | bacterial abc transporter protein | *artI* | 438 | ZP04797502 | 6.93E-061 | 100 | 187.96 | 109 | 109 | 7 |
| 24 | gi\|373498518\|ref\|ZP_09589027.1\|nickel ABC transporter, permease subunit NikB | binding-protein-dependent transport system inner membrane component | *nikB* | 139 | ZP09589027 | 2.29E-004 | 71 | 33.11 | 28 | 20 | 1 |
| 25 | gi\|57866871\|ref\|YP_188555.1\|branched-chain amino acid ABC transporter substrate-binding protein | branched-chain amino acid transport system ii carrier protein | *brnQ* | 442 | YP188555 | 4.57E-077 | 100 | 232.65 | 143 | 143 | 2 |
| 26 | gi\|27467140\|ref\|NP_763777.1\|glycine betaine/carnitine/choline ABC transporter ATP-binding opuCA | choline abcatp-binding protein | *opuBA* | 241 | NP763777 | 3.50E-048 | 100 | 154.45 | 76 | 76 | 11 |
| 27 | gi\|242242381\|ref\|ZP_04796826.1\|ABC superfamily ATP binding cassette transporter, membrane protein | cobalt abcpermease protein | *cbiQ* | 148 | ZP04796826 | 6.88E-018 | 100 | 71.25 | 33 | 33 | 1 |
| 28 | gi\|271966885\|ref\|YP_003341081.1\|unnamed protein product | conserved hypothetical dehydratase | *MMAR_4009* | 168 | YP003341081 | 1.07E-005 | 60 | 35.81 | 35 | 21 | 1 |
| 29 | gi\|157737350\|ref\|YP_001490033.1\|iron compound ABC transporter, permease | crispr-associated protein cas1 | *ygbT* | 153 | YP001490033 | 9.13E-005 | 61 | 34.65 | 39 | 24 | 1 |
| 30 | gi\|258445628\|ref\|ZP_05693808.1\|ABC transporter | d-methionine transport system permease protein | *metI* | 110 | ZP05693808 | 2.52E-004 | 100 | 32.73 | 36 | 36 | 1 |
| 31 | gi\|116669747\|ref\|YP_830680.1\|unnamed protein product | dipeptide abc transporter substrate-binding protein | *dppA* | 154 | YP830680 | 0 | 63 | 30.8 | 41 | 26 | 1 |
| 32 | gi\|365230108\|gb\|EHM71226.1\|efflux ABC transporter, permease protein | efflux abcpermease protein | *macB_7* | 123 | EHM71226 | 8.45E-016 | 100 | 65.08 | 32 | 32 | 4 |
| 33 | gi\|365225081\|gb\|EHM66334.1\|ferrichrome ABC transporter, ATP-binding protein FhuC | ferrichrome transport atp-binding protein | *fhuC* | 404 | EHM66334 | 1.98E-047 | 100 | 151.37 | 77 | 77 | 6 |
| 34 | gi\|16080434\|ref\|NP_391261.1\|glycine betaine/carnitine/choline/choline sulfate ABC transporter osmoprotectant-binding lipoprotein | glycine betainecarnitine choline-binding protein | *opuCC* | 117 | NP391261 | 8.65E-007 | 91 | 40.05 | 23 | 21 | 2 |
| 35 | gi\|323359089\|ref\|YP_004225485.1\|phosphate ABC transporter ATPase | inosine-uridine preferring nucleoside hydrolase | *PSF113_2010* | 109 | YP004225485 | 0 | 60 | 29.65 | 35 | 21 | 1 |
| 36 | gi\|365225051\|gb\|EHM66304.1\|iron chelate uptake ABC transporter, FeCT family, permease protein | iron chelate uptake abcpermeaseprotein | *sirC* | 170 | EHM66304 | 4.42E-027 | 100 | 96.29 | 47 | 47 | 5 |
| 37 | gi\|242241996\|ref\|ZP_04796441.1\|ABC superfamily ATP binding cassette transporter, binding protein | manganese abc transporter substrate-binding lipoprotein | *psaA* | 1660 | ZP04796441 | 2.00E-139 | 94 | 403.68 | 220 | 208 | 3 |
| 38 | gi\|365232243\|gb\|EHM73250.1\|metal ion ABC transporter, permease protein | manganese iron transport system permease | *sitD* | 275 | EHM73250 | 2.35E-008 | 100 | 47.37 | 22 | 22 | 1 |
| 39 | gi\|165918414\|ref\|ZP_02218500.1\|D-methionine ABC transporter, ATP-binding protein | methionine transport atp-binding protein | *metN1* | 119 | ZP02218500 | 0 | 72 | 30.03 | 29 | 21 | 1 |
| 40 | gi\|71908396\|ref\|YP_285983.1\|molybdate ABC transporter permease | molybdateabc inner membrane subunit | *modC* | 153 | YP285983 | 6.30E-005 | 60 | 35.42 | 38 | 23 | 1 |
| 41 | gi\|319400648\|gb\|EFV88873.1\|modA protein | molybdateabcperiplasmicmolybdate-binding protein | *modA* | 142 | EFV88873 | 1.80E-018 | 100 | 69.32 | 34 | 34 | 4 |
| 42 | gi\|329731217\|gb\|EGG67587.1\|molybdate ABC transporter, permease protein | molybdateabcpermease protein | *modB* | 268 | EGG67587 | 2.29E-034 | 100 | 115.55 | 80 | 80 | 2 |
| 43 | gi\|317152696\|ref\|YP_004120744.1\|nickel ABC transporter substrate-binding protein | nickel abc transporter substrate-binding protein | *nikA* | 122 | YP004120744 | 0.01 | 62 | 30.42 | 29 | 18 | 2 |
| 44 | gi\|302532457\|ref\|ZP_07284799.1\|nitrate ABC transporter, permease | nitrate abcpermease | *RRSWK_01811* | 163 | ZP07284799 | 1.55E-005 | 55 | 35.42 | 47 | 26 | 1 |
| 45 | gi\|282875516\|ref\|ZP_06284387.1\|putative oligopeptide ABC transporter, permease protein OppC | oligopeptideabcpermease protein | *oppB* | 136 | ZP06284387 | 5.07E-019 | 100 | 73.17 | 36 | 36 | 3 |
| 46 | gi\|327438710\|dbj\|BAK15075.1\|ABC-type oligopeptide transport system, ATPase component | oligopeptideabc transporter atp-binding protein | *amiF* | 201 | BAK15075 | 0 | 65 | 31.96 | 29 | 19 | 3 |
| 47 | gi\|365811626\|gb\|AEW99841.1\|oligopeptide/dipeptide ABC transporter, ATPase subunit | peptidase caspase catalytic subunit p20 | *Sfum_0556* | 138 | AEW99841 | 6.25E-004 | 53 | 31.57 | 43 | 23 | 1 |
| 48 | gi\|319400396\|gb\|EFV88630.1\|phosphate/phosphite/phosphonate ABC transporter, periplasmic binding protein | phosphate phosphitephosphonateabcperiplasmic binding protein | *phnD* | 122 | EFV88630 | 2.90E-020 | 97 | 76.64 | 39 | 38 | 1 |
| 49 | gi\|291298064\|ref\|YP_003509342.1\|ABC transporter-like protein | sensor protein | *zraS* | 186 | YP003509342 | 7.25E-005 | 46 | 32.73 | 41 | 19 | 1 |
| 50 | gi\|359683203\|ref\|ZP_09253204.1\|ABC transporter permease | serine threonine protein kinase | *pknB* | 109 | ZP09253204 | 8.61E-004 | 59 | 30.8 | 32 | 19 | 1 |
| 51 | gi\|365231674\|gb\|EHM72701.1\|ABC transporter, solute-binding protein | spermidineputrescineabc superfamily atp binding cassette binding protein | *potA* | 168 | EHM72701 | 4.48E-017 | 100 | 69.32 | 31 | 31 | 1 |
| 52 | gi\|319891892\|ref\|YP_004148767.1\|hypothetical protein SPSINT_0602 | sugar abc transporter substrate-binding protein | *ypdA* | 171 | YP004148767 | 9.52E-008 | 74 | 38.89 | 31 | 23 | 1 |
| 53 | gi\|365225076\|gb\|EHM66329.1\|ABC transporter, ATP-binding protein | teichoic acids export protein atp-binding subunit | *tagH* | 104 | EHM66329 | 1.78E-010 | 100 | 46.21 | 22 | 22 | 1 |
| 54 | gi\|357415095\|ref\|YP_004926831.1\|iron ABC transporter permease | transport system permease protein | *irtA* | 147 | YP004926831 | 2.86E-004 | 57 | 32.73 | 40 | 23 | 1 |
