# Supplementary material for: De novo transcriptome analysis of halotolerant bacterium Staphylococcus sp. strain P-TSB-70 isolated from East coast of India: In search of salt stress tolerant genes
Source: PLoS One. 2020 Feb 10;15(2):e0228199. doi: 10.1371/journal.pone.0228199 (PMC7010390; doi:10.1371/journal.pone.0228199)
Supplement: S3 Table — (DOCX) [file pone.0228199.s010.docx]

**S3 Table. List of upregulated sodium antiporter genes unique to *Staphylococcus* sp. in response to salt stress**

| **Sl no.** | **Gene ID** | **Functional annotation** | **Gene** | **Sequence length** | **Hit accession** | **E-Value** | **Similarity** | **Score** | **Alignment length** | **Positives** | **Sequence similar to functional target genes** |
| --- | --- | --- | --- | --- | --- | --- | --- | --- | --- | --- | --- |
| 1 | gi\|242242052\|ref\|ZP04796497.1\|  DASS family divalent anion:sodium (Na+) symporter | anion transporter family protein | *citT* | 117 | ZP04796497 | 1.86E-014 | 100 | 61.62 | 28 | 28 | 1 |
| 2 | gi\|242241512\|ref\|ZP04795957.1\|  F-ATPase superfamily proton (H+)- or sodium (Na+)-translocating F family ATPase delta subunit | atp synthase delta subunit | *atpH* | 476 | ZP04795957 | 8.19E-060 | 100 | 179.49 | 93 | 93 | 1 |
| 3 | gi\|27468617\|ref\|NP765254.1\|  ATP synthase F0F1 subunit epsilon | atp synthase epsilon subunit | *atpC* | 284 | NP765254 | 5.35E-050 | 100 | 151.37 | 79 | 79 | 1 |
| 4 | gi\|314935597\|ref\|ZP07842949.1\|  putative Na+/H+ antiporter | cpa2 family | *HMPREF9550_02505* | 134 | ZP07842949 | 9.34E-009 | 92 | 46.21 | 26 | 24 | 1 |
| 5 | gi\|151221032\|ref\|YP001331854.1\|  putative monovalent cation/H+ antiporter subunit C | monovalent cation H+ antiporter subunit c | *mrpC* | 696 | YP001331854 | 3.29E-057 | 89 | 175.64 | 132 | 118 | 2 |
| 6 | gi\|242241995\|ref\|ZP04796440.1\|  CPA1 family monovalent cation (K+ or Na+):proton (H+) antiporter-1 | Na+ H+ antiporter | *SO_2537* | 574 | ZP04796440 | 1.78E-114 | 98 | 337.81 | 177 | 175 | 15 |
| 7 | gi\|365231395\|gb\|EHM72441.1\|  Na+/H+ antiporter family protein | Na+ H+ antiporter family protein | *nhaA* | 304 | EHM72441 | 4.33E-046 | 96 | 149.83 | 86 | 83 | 14 |
| 8 | gi\|319401281\|gb\|EFV89493.1\|  Na+ dependent nucleoside transporter family protein | purine nucleoside transport protein | *nupG* | 205 | EFV89493 | 5.19E-027 | 100 | 95.9 | 47 | 47 | 4 |
