# Supplementary material for: De novo transcriptome analysis of halotolerant bacterium Staphylococcus sp. strain P-TSB-70 isolated from East coast of India: In search of salt stress tolerant genes
Source: PLoS One. 2020 Feb 10;15(2):e0228199. doi: 10.1371/journal.pone.0228199 (PMC7010390; doi:10.1371/journal.pone.0228199)
Supplement: S4 Table — (DOCX) [file pone.0228199.s011.docx]

**S4 Table. List of upregulated sodium symporter genes unique to *Staphylococcus* sp. in response to salt stress**

| **Sl no.** | **Gene ID** | **Functional annotation** | **Gene** | **Sequence length** | **Hit accession** | **E-Value** | **Similarity** | **Score** | **Alignment length** | **Positives** | **Sequence similar to functional target genes** |
| --- | --- | --- | --- | --- | --- | --- | --- | --- | --- | --- | --- |
| 1 | gi\|365231182\|gb\|EHM72240.1\|  sodium/prolinesymporter | sodium prolinesymporter | *putP* | 208 | EHM72240 | 3.03E-029 | 100 | 105.92 | 52 | 52 | 5 |
| 2 | gi\|242242052\|ref\|ZP04796497.1\|  DASS family divalent anion:sodium (Na+) symporter | dass family | *RradSPS_0118* | 336 | ZP04796497 | 7.23E-016 | 98 | 64.31 | 59 | 58 | 1 |
| 3 | gi\|303229246\|ref\|ZP07316042.1\|  transporter, divalent anion:Na+ symporter (DASS) family protein | 2-oxoglutarate malate translocator | *2486* | 111 | ZP07316042 | 5.00E-004 | 47 | 32.34 | 34 | 16 | 1 |
| 4 | gi\|27469244\|ref\|NP765881.1\|  sodium-dependent transporter | sodium:neurotransmittersymporter family protein | *JGI23_00702* | 174 | NP765881 | 5.12E-016 | 100 | 65.08 | 33 | 33 | 5 |
| 5 | gi\|242242052\|ref\|ZP04796497.1\|  DASS family divalent anion:sodium (Na+) symporter | anion transporter family protein | *BN812_01036* | 117 | ZP04796497 | 1.86E-014 | 100 | 61.62 | 28 | 28 | 1 |
| 6 | gi\|365232378\|gb\|EHM73377.1\|  transporter, dicarboxylate/amino acid:cation Na+/H+ symporter family protein | dicarboxylate amino acid:cation Na+ H+ symporter family protein | *sstT* | 212 | EHM73377 | 1.73E-035 | 100 | 121.71 | 62 | 62 | 4 |
| 7 | gi\|365224940\|gb\|EHM66197.1\|  sodium/glutamate symporter | sodium glutamate symporter | *gltS* | 222 | EHM66197 | 2.82E-031 | 100 | 109 | 52 | 52 | 2 |
| 8 | gi\|27467362\|ref\|NP763999.1\|  sugar efflux transporter | sugar efflux transporter | *setA* | 283 | NP763999 | 9.87E-049 | 88 | 155.22 | 94 | 83 | 1 |
| 9 | gi\|242243607\|ref\|ZP04798051.1\|  SSS family solute:sodium (Na+) symporter | sss family | *SsS58_08188* | 128 | ZP04798051 | 2.81E-020 | 97 | 73.56 | 39 | 38 | 1 |
| 10 | gi\|242243303\|ref\|ZP04797748.1  \|hypothetical protein HMPREF07911829 | daacs family dicarboxylate amino acid:sodium (Na+) symporter | *FD50_GL000567* | 164 | ZP04797748 | 1.50E-015 | 100 | 60.85 | 29 | 29 | 1 |
| 11 | gi\|365227111\|gb\|EHM68314.1\|  transporter, dicarboxylate/amino acid:cation (Na+ or H+) symporter (DAACS) domain protein | proton sodium-glutamate symport protein | *gltT* | 198 | EHM68314 | 1.86E-020 | 88 | 77.03 | 45 | 40 | 2 |
| 13 | gi\|371772855\|gb\|EHO57480.1\|  hypothetical protein PredeDRAFT1102 | branched chain amino acid:cationsymporter family protein | *HMPREF3211_01754* | 215 | EHO57480 | 1.65E-006 | 79 | 38.12 | 48 | 38 | 1 |
| 14 | gi\|365237189\|gb\|EHM78046.1\|  sodium:alaninesymporter domain protein, partial | amino acid carrier protein | *acpP* | 184 | EHM78046 | 2.16E-025 | 82 | 88.97 | 58 | 48 | 1 |
| 15 | gi\|365232378\|gb\|EHM73377.1\|  transporter, dicarboxylate/amino acid:cation Na+/H+ symporter family protein | sodium:dicarboxylatesymporter family protein | *sdcS* | 304 | EHM73377 | 2.72E-043 | 96 | 144.44 | 89 | 86 | 1 |
