# Supplementary material for: De novo transcriptome analysis of halotolerant bacterium Staphylococcus sp. strain P-TSB-70 isolated from East coast of India: In search of salt stress tolerant genes
Source: PLoS One. 2020 Feb 10;15(2):e0228199. doi: 10.1371/journal.pone.0228199 (PMC7010390; doi:10.1371/journal.pone.0228199)
Supplement: S5 Table — (DOCX) [file pone.0228199.s012.docx]

**S5 Table. List of upregulated glycine-betainegenes unique to *Staphylococcus* sp. in response to salt stress**

| **Sl no.** | **Gene ID** | **Functional annotation** | **Gene** | **Sequence length** | **Hit accession** | **E-Value** | **Similarity** | **Score** | **Alignment Length** | **Positives** | **Sequence similar to functional target genes** |
| --- | --- | --- | --- | --- | --- | --- | --- | --- | --- | --- | --- |
| 1 | gi\|72384791\|gb\|AAZ67687.1\|  glycine betaine aldehyde dehydrogenase putative | betaine aldehyde dehydrogenase | *betB* | 264 | AAZ67687 | 8.52E-026 | 82 | 95.9 | 70 | 58 | 1 |
| 2 | gi\|242243486\|ref\|ZP04797931.1\|  BCCT family betaine transporter | betainecarnitine choline family | *opuCB* | 1300 | ZP04797931 | 0 | 96 | 751.9 | 425 | 412 | 6 |
| 3 | gi\|72384791\|gb\|AAZ67687.1\|  glycine betaine aldehyde dehydrogenase putative | betaine-aldehyde dehydrogenase | *betB* | 516 | AAZ67687 | 2.60E-111 | 94 | 320.86 | 171 | 162 | 5 |
| 4 | gi\|27467140\|ref\|NP763777.1\|  glycine betaine/carnitine/choline ABC transporter ATP-binding opuCA | choline abcatp-binding protein | *proV_1* | 241 | NP763777 | 3.50E-048 | 100 | 154.45 | 76 | 76 | 1 |
| 5 | gi\|365878078\|ref\|ZP09417566.1\|  choline/carnitine/betaine transporter | choline carnitinebetaine transporter | *betP_2* | 114 | ZP09417566 | 2.97E-004 | 76 | 29.26 | 30 | 23 | 1 |
| 6 | gi\|242243766\|ref\|ZP04798210.1\|  glycine betaine/choline ABC superfamily ATP binding cassette transporter, membrane protein | choline transport system permease protein opubb | *opuBB* | 561 | ZP04798210 | 4.23E-038 | 94 | 127.49 | 87 | 82 | 1 |
| 7 | gi\|57636194\|gb\|AAW52982.1\|  choline/carnitine/betaine transporter | choline transporter | *betT* | 382 | AAW52982 | 8.06E-061 | 100 | 192.59 | 115 | 115 | 2 |
| 8 | gi\|365228580\|gb\|EHM69761.1\|  glycine betaine/carnitine/choline transport system permease protein opuCD | glycine betainecarnitine choline transport system permease protein opucd | *opuCD* | 298 | EHM69761 | 6.59E-032 | 100 | 109.77 | 58 | 58 | 7 |
| 9 | gi\|319400451\|gb\|EFV88685.1\|  glycine/betaine/carnitine/choline-binding protein | glycine betainecarnitine choline-binding protein | *opuCC* | 271 | EFV88685 | 3.67E-047 | 100 | 150.21 | 75 | 75 | 8 |
| 10 | gi\|291440664\|ref\|ZP06580054.1\|  glycine betaine transport system permease protein | glycine betaine transport system permease protein | *opuAB* | 120 | ZP06580054 | 2.18E-004 | 53 | 30.03 | 39 | 21 | 1 |
| 11 | gi\|57866157\|ref\|YP187819.1\|  major facilitator family transporter | prolinebetaine transporter | *proP* | 1141 | YP187819 | 1.78E-102 | 98 | 308.53 | 173 | 171 | 20 |
