# Supplementary material for: De novo transcriptome analysis of halotolerant bacterium Staphylococcus sp. strain P-TSB-70 isolated from East coast of India: In search of salt stress tolerant genes
Source: PLoS One. 2020 Feb 10;15(2):e0228199. doi: 10.1371/journal.pone.0228199 (PMC7010390; doi:10.1371/journal.pone.0228199)
Supplement: S6 Table — (DOCX) [file pone.0228199.s013.docx]

**S6 Table. List of upregulated trehalose genes unique to *Staphylococcus* sp. in response to salt stress**

| **Sl no.** | **Gene ID** | **Functional annotation** | **Gene** | **Sequence length** | **Hit accession** | **E-Value** | **Similarity** | **Score** | **Alignment length** | **Positives** | **Sequence similar to functional target genes** |
| --- | --- | --- | --- | --- | --- | --- | --- | --- | --- | --- | --- |
| 1 | gi\|324999767\|ref\|ZP08120879.1\|  malto-oligosyltrehalosetrehalohydrolase | malto-oligosyltrehalosetrehalohydrolase | *treZ* | 117 | ZP08120879 | 0.01 | 52 | 29.26 | 34 | 18 | 1 |
| 2 | gi\|373481951\|ref\|ZP09572760.1\|  maltooligosyltrehalose hydrolase | maltooligosyltrehalose hydrolase | *treZ-1* | 107 | ZP09572760 | 0 | 62 | 29.26 | 29 | 18 | 1 |
