# Supplementary material for: De novo transcriptome analysis of halotolerant bacterium Staphylococcus sp. strain P-TSB-70 isolated from East coast of India: In search of salt stress tolerant genes
Source: PLoS One. 2020 Feb 10;15(2):e0228199. doi: 10.1371/journal.pone.0228199 (PMC7010390; doi:10.1371/journal.pone.0228199)
Supplement: S7 Table — (DOCX) [file pone.0228199.s014.docx]

**S7 Table. Classification of the gene ontology study of the functionally annotated genes**

| **Sl no.** | **Functional annotation** | **GO biological process** | **GO cellular component** | **GO Molecular Function** |
| --- | --- | --- | --- | --- |
| 1 | abc transporter | sodium ion transport; transmembrane transport | membrane | transporter activity |
| 2 | Sodium:neurotransmittersymporter family protein | neurotransmitter transport | integral to plasma membrane | neurotransmitter:sodiumsymporter activity |
| 3 | sodium prolinesymporter | transmembrane transport; proline transport; sodium ion transport | plasma membrane; integral to membrane | sodium ion binding; proline:sodiumsymporter activity |
| 4 | Na+ H+ antiporter | regulation of pH; sodium ion transport | integral to membrane | sodium:hydrogenantiporter activity |
| 5 | dass family | sodium ion transport; transmembrane transport | membrane | transporter activity |
| 6 | dicarboxylate amino acid:cation Na+ H+ symporter family protein | dicarboxylic acid transport | integral to membrane | sodium:dicarboxylatesymporter activity |
| 7 | amino acid carrier protein | sodium ion transport | membrane | sodium:amino acid symporter activity |
| 8 | betaine-aldehyde dehydrogenase | betaine biosynthetic process; oxidation reduction | - | betaine-aldehyde dehydrogenase activity |
| 9 | proton sodium-glutamate symporter protein | dicarboxylic acid transport | plasma membrane; integral to membrane | sodium:dicarboxylatesymporter activity |
| 10 | sodium glutamate symporter | L-glutamate transport | integral to membrane | glutamate:sodiumsymporter activity |
