# Supplementary material for: De novo transcriptome analysis of halotolerant bacterium Staphylococcus sp. strain P-TSB-70 isolated from East coast of India: In search of salt stress tolerant genes
Source: PLoS One. 2020 Feb 10;15(2):e0228199. doi: 10.1371/journal.pone.0228199 (PMC7010390; doi:10.1371/journal.pone.0228199)
Supplement: S8 Table — (DOCX) [file pone.0228199.s015.docx]

**S8 Table. List of upregulated genes associated with salt tolerant pathways**

| **Sl no.** | **Functional annotation** | **Gene** | **Related pathway** |
| --- | --- | --- | --- |
| 1 | abc 3 transport family protein | *abc3* | ATP binding |
| 2 | abc atp-binding protein | *Rv1747* | ATP binding |
| 3 | abc permease protein | *abc13p* | Cellular component |
| 4 | abc quaternary amine uptake transporter substrate-binding protein | *HMPREF1110_0377* | Transporter activity |
| 5 | abc solute-binding protein | *TCCBUS3UF1_15170* | Amino acid-transporting ATPase activity |
| 6 | abc substrate-binding family 3 | *ECBG_00399* | Metal ion binding |
| 7 | abc transporter | *yojI* | ATP binding |
| 8 | abc-2 type transporter | *GLRG_09770* | ATPase activity, coupled to transmembrane movement of substances |
| 9 | branched-chain amino acid transport system ii carrier protein | *brnQ* | Component of the transport system for branched-chain amino acids |
| 10 | conserved hypothetical dehydratase | *MMAR_4009* | Catalytic activity |
| 11 | dipeptide abc transporter substrate-binding protein | *dppA* | Transmembrane transport |
| 12 | glycine betaine carnitine choline-binding protein | *opuCC* | Quaternary-ammonium-compound-transporting ATPase activity |
| 13 | inosine-uridine preferring nucleoside hydrolase | *PSF113_2010* | Purine nucleosidaseactvity |
| 14 | iron chelate uptake abc permease protein | *sirC* | Precorrn-2 dehydrogenase activity |
| 15 | manganese abc transporter substrate-binding lipoprotein | *psaA* | Oxidoreductase |
| 16 | peptidase caspase catalytic subunit p20 | *Sfum_0556* | Cysteine-type endopeptidase activity |
| 17 | phosphate phosphate phosphonate abc periplasmic binding protein | *phnD* | Transmembrane transport |
| 18 | sensor protein | *zraS* | ATP binding |
| 19 | serine threonine protein kinase | *pknB* | ATP binding |
| 20 | spermidineputrescineabc superfamily atp binding cassette binding protein | *potA* | ATP binding |
| 21 | sugar abc transporter substrate-binding protein | *ypdA* | ATP binding |
| 22 | teichoic acids export protein atp-binding subunit | *tagH* | ATP bindng |
| 23 | transport system permease protein | *irtA* | ATPase ativity, coupled to transmembrane movement of substances |
| 24 | betaine aldehyde dehydrogenase | *betB* | Betaine-aldehyde dehydrogenase activity |
| 25 | betainecarnitine choline family | *opuCB* | Quaternary-ammonium-compound-transporting ATPase activity |
| 26 | choline abcatp-binding protein | *proV_1* | ATP binding |
| 27 | choline carnitinebetaine transporter | *betP_2* | Transporter activity |
| 28 | choline transport system permease protein opubb | *opuBB* | Transporter activity |
| 29 | choline transporter | *betT* | Transorter activity |
| 30 | glycine betainecarnitine choline transport system permease protein opucd | *opuCD* | Quaternary-ammonium-compound-transporting ATPase activity |
| 31 | anion transporter family protein | *citT* | Transporter activity |
| 32 | atp synthase delta subunit | *atpH* | Proton-transporting ATP synthase activity, rotational mechanism |
| 33 | atp synthase epsilon subunit | *atpC* | Proton-transporting ATP synthase activity, rotational mechanism |
| 34 | cpa2 family | *HMPREF9550_02505* | Solute:protonantiporter activity |
| 35 | monovalent cation H+ antiporter subunit c | *mrpC* | Antiporter activity |
| 36 | Na+ H+ antiporter | *SO_2537* | Solute:protonantiporter activity |
| 37 | purine nucleoside transport protein | *nupG* | Cytidinetransmembrane transporter activity |
| 38 | sodium prolinesymporter | *putP* | Proline:sodiumsymporter activity |
| 39 | dass family | *RradSPS_0118* | Transporter activity |
| 40 | 2-oxoglutarate malate translocator | *2486* | Transporter activity |
| 41 | sodium:neurotransmitter symporter family protein | *JGI23_00702* | Neurotransmitter:sodiumsymporter activity |
| 42 | dicarboxylate amino acid:cation Na+ H+ symporter family protein | *sstT* | Neutral amino acid:sodiumsymporter activity |
| 43 | sodium glutamate symporter | *gltS* | Glutamate:sodiumsymporter activity |
| 44 | sugar efflux transporter | *setA* | Sugar efflux transmembrane transporter activity |
| 45 | sss family | *SsS58_08188* | DNA binding |
| 46 | daacs family dicarboxylate amino acid:sodium (Na+) symporter | *FD50_GL000567* | Symporter activity |
| 47 | proton sodium-glutamate symport protein | *gltT* | Symporter activity |
| 48 | branched chain amino acid:cationsymporter family protein | *HMPREF3211_01754* | Transporter activity |
| 49 | amino acid carrier protein | *acpP* | Acyl binding |
| 50 | sodium:dicarboxylatesymporter family protein | *sdcS* | Symporter activity |
| 51 | malto-oligosyltrehalosetrehalohydrolase | *treZ* | Trehalose biosynthetic process |
| 52 | maltooligosyltrehalose hydrolase | *treZ-1* | Trehalose biosynthetic process |
