# Supplementary material for: De novo transcriptome analysis of halotolerant bacterium Staphylococcus sp. strain P-TSB-70 isolated from East coast of India: In search of salt stress tolerant genes
Source: PLoS One. 2020 Feb 10;15(2):e0228199. doi: 10.1371/journal.pone.0228199 (PMC7010390; doi:10.1371/journal.pone.0228199)
Supplement: S9 Table — (DOCX) [file pone.0228199.s016.docx]

**S9 Table. List of upregulated genes associated with different metabolic pathways**

| **Sl no.** | **Functional annotation** | **Gene** | **Related pathway** |
| --- | --- | --- | --- |
|  | succinate- beta subunit | *sdhB* | [succinate dehydrogenase (ubiquinone) activity](http://www.ebi.ac.uk/QuickGO/GTerm?id=GO:0008177) |
|  | succinate-semialdehyde dehydrogenase | *sad* | [succinate-semialdehyde dehydrogenase (NAD+) activity](http://www.ebi.ac.uk/QuickGO/GTerm?id=GO:0004777) |
|  | succinate dehydrogenase iron-sulfur protein subunit | *sdhB* | [metal ion binding](http://www.ebi.ac.uk/QuickGO/GTerm?id=GO:0046872) |
|  | succinate cytochrome b556 subunit | *sdhC* | Oxidation reduction |
|  | adenylosuccinatelyase | *purB* | Purine ribonucleotide biosynthetic process |
|  | argininosuccinatelyase | *argH* | Arginine biosynthetic process via ornithine |
|  | cyanate transport protein | *cynX* | Transmembrane transporter |
|  | riboflavin transporter | *ribU* | [riboflavin transporter activity](http://www.ebi.ac.uk/QuickGO/GTerm?id=GO:0032217) |
|  | fructose-bisphosphate class i | *fbaB* | [fructose-bisphosphatealdolase activity](http://www.ebi.ac.uk/QuickGO/GTerm?id=GO:0004332) |
|  | competence factor transporting permease atp-binding protein | *comA* | [ATPase activity, coupled to transmembrane movement of substances](http://www.ebi.ac.uk/QuickGO/GTerm?id=GO:0042626) |
|  | sugar phosphate antiporter | *uhpT* | [hexose phosphate transport](http://www.ebi.ac.uk/QuickGO/GTerm?id=GO:0015712) |
|  | sugar efflux transporter | *setB* | [sugar efflux transmembrane transporter activity](http://www.ebi.ac.uk/QuickGO/GTerm?id=GO:0015542) |
|  | low quality protein: glucose-6-phosphate isomerase | *pgi* | [glucose-6-phosphate isomerase activity](http://www.ebi.ac.uk/QuickGO/GTerm?id=GO:0004347) |
|  | chemotaxis sensor histidine kinase | *CheA* | Phosphorelay sensor kinase activity |
|  | response regulator receiver modulated metal dependent phosphohydrolase | *ThidrDRAFT_4290* | Phosphorelay signal transduction system |
|  | di-and tricarboxylate transporters | *MHY-13230* | Transmembrane transporter |
|  | glucose-methanol-choline oxidoreductase:nad binding site | *BAB1_0577* | Choline dehydrogenase activity |
|  | glucose-1-phosphate adenylyl transferase | *glgC* | [glucose-1-phosphate adenylyltransferase activity](http://www.ebi.ac.uk/QuickGO/GTerm?id=GO:0008878) |
|  | glucose-1-phosphate thymidylyl transferase | *rfbA* | [glucose-1-phosphate thymidylyltransferase activity](http://www.ebi.ac.uk/QuickGO/GTerm?id=GO:0008879) |
|  | pas pac sensor hybrid histidine kinase | *RGE_17160* | Phosphorelay sensor kinase activity |
|  | glycerol-3-phosphate o-acyltransferase | *plsB* | [glycerol-3-phosphate O-acyltransferase activity](http://www.ebi.ac.uk/QuickGO/GTerm?id=GO:0004366) |
|  | cobalt transporter subunit | *corA* | Cobalt ion transmembrane transporter activity |
|  | phosphate dikinase | *PPDK1* | Kinase activity |
|  | 4-methyl-5(b-hydroxyethyl)-thiazole monophosphate biosynthesis enzyme | *P0665D10.15* | Glyoxylase III activity |
|  | thiamine phosphate synthase | *thiE* | [thiamine-phosphate diphosphorylase activity](http://www.ebi.ac.uk/QuickGO/GTerm?id=GO:0004789) |
|  | chemotaxis-specific histidine kinase | *cheA* | Phosphorelay sensor kinase activity |
|  | low quality protein: two component system response regulator | *ARR12* | [phosphorelay response regulator activity](http://www.ebi.ac.uk/QuickGO/GTerm?id=GO:0000156) |
|  | succinate dehydrogenase flavoprotein subunit | *sdhA* | [succinate dehydrogenase (ubiquinone) activity](http://www.ebi.ac.uk/QuickGO/GTerm?id=GO:0008177) |
|  | anaerobic c4-dicarboxylate membrane | *dcuA* | [C4-dicarboxylate transmembrane transporter](http://www.ebi.ac.uk/QuickGO/GTerm?id=GO:0015556) |
|  | pas pac sensor signal transduction histidine kinase | *dhkA* | [transmembrane receptor histidine kinase activity](http://www.ebi.ac.uk/QuickGO/GTerm?id=GO:0009784) |
|  | adenosylcobinamide kinase adenosylcobinamide phosphate | *cobY* | [cobinamide phosphate guanylyltransferase activity](http://www.ebi.ac.uk/QuickGO/GTerm?id=GO:0008820) |
|  | guanylyl transferase | *mobA* | [GTP binding](http://www.ebi.ac.uk/QuickGO/GTerm?id=GO:0005525) |
|  | possible filamentous hemagglutinin outer membrane protein | *fhaC* | [porin activity](http://www.ebi.ac.uk/QuickGO/GTerm?id=GO:0015288) |
|  | pyridoxamine 5 -phosphate oxidase-related fmn-binding protein | *RL3536* | [oxidoreductase activity](http://www.ebi.ac.uk/QuickGO/GTerm?id=GO:0016491) |
|  | dmt superfamily drug metabolite transporter | *emrE* | [drug:protonantiporter activity](http://www.ebi.ac.uk/QuickGO/GTerm?id=GO:0015307) |
|  | phosphonate-transporting atpase | *BrE312_0915* | [organic phosphonatetransmembrane-transporting ATPase activity](http://www.ebi.ac.uk/QuickGO/GTerm?id=GO:0015416) |
|  | citrate transporter | *citA* | [citrate metabolic process](http://www.ebi.ac.uk/QuickGO/GTerm?id=GO:0006101) |
|  | n-acetyl-gamma-glutamyl-phosphate reductase | *At2g19940* | [N-acetyl-gamma-glutamyl-phosphate reductase activity](http://www.ebi.ac.uk/QuickGO/GTerm?id=GO:0003942) |
|  | sugar efflux transporter | *SWEET16* | [fructose transmembrane transporter activity](http://www.ebi.ac.uk/QuickGO/GTerm?id=GO:0005353) |
|  | electron transport complex protein | *cyoB* | [cytochrome bo3 ubiquinol oxidase activity](http://www.ebi.ac.uk/QuickGO/GTerm?id=GO:0009486) |
|  | periplasmic sensor signal transduction histidine kinase | *baeS* | [phosphorelay signal transduction system](http://www.ebi.ac.uk/QuickGO/GTerm?id=GO:0000160) |
|  | oligopeptide transport system permease protein | *oppB* | [oligopeptide-transporting ATPase activity](http://www.ebi.ac.uk/QuickGO/GTerm?id=GO:0015421) |
|  | 1-acylglycerol-3-phosphate o-acyltransferase | *At4g24160* | [carboxylic ester hydrolase activity](http://www.ebi.ac.uk/QuickGO/GTerm?id=GO:0052689) |
|  | phosphate dikinase | *ppdK* | [pyruvate, phosphate dikinase activity](http://www.ebi.ac.uk/QuickGO/GTerm?id=GO:0050242) |
|  | actinorhodin transporter | *actII-2* | [response to antibiotic](http://www.ebi.ac.uk/QuickGO/GTerm?id=GO:0046677) |
|  | teichoic-acid-transporting atpase | *LLCRE1631_01548* | [teichoic acid transport](http://www.ebi.ac.uk/QuickGO/GTerm?id=GO:0015777) |
|  | respiratory nitrate reductase protein | *narG* | [nitrate assimilation](http://www.ebi.ac.uk/QuickGO/GTerm?id=GO:0042128) |
|  | rnd family efflux transporter mfp subunit | *PFL_4081* | [protein transporter activity](http://www.ebi.ac.uk/QuickGO/GTerm?id=GO:0008565) |
|  | fructose-1-phosphate kinase | *pfkB* | [1-phosphofructokinase activity](http://www.ebi.ac.uk/QuickGO/GTerm?id=GO:0008662) |
|  | 4-methyl-5(b-hydroxyethyl)-thiazole monophosphate biosynthesis protein | *PRCDC_0625900* | [hydroxyethylthiazole kinase activity](http://www.ebi.ac.uk/QuickGO/GTerm?id=GO:0004417) |
|  | low quality protein: ni2+-co2+ transporter family | *CAMGR0001_2838* | [nickel cationtransmembrane transporter activity](http://www.ebi.ac.uk/QuickGO/GTerm?id=GO:0015099) |
|  | f-type h+-transporting atpase subunit b | *atpF* | [hydrogen ion transmembrane transporter activity](http://www.ebi.ac.uk/QuickGO/GTerm?id=GO:0015078) |
|  | pas pac sensor hybrid histidine kinase | *RGE_17160* | [phosphorelay sensor kinase activity](http://www.ebi.ac.uk/QuickGO/GTerm?id=GO:0000155) |
|  | undecaprenyl-phosphate n-acetylglucosaminyl 1-phosphate transferase | *wecA* | Phospho-N-acetylmuramoyl-pentapeptide-transferase activity |
|  | electron transport g subunit | *PUT1* |  |
|  | apc family amino acid-polyamine-organocation transporter | *arcD* | [arginine:ornithineantiporter activity](http://www.ebi.ac.uk/QuickGO/GTerm?id=GO:0043858) |
|  | Fumarate lyase | *fumC* | [fumaratehydratase activity](http://www.ebi.ac.uk/QuickGO/GTerm?id=GO:0004333) |
|  | nodulation abc transporter | *nodI* | [transporter activity](http://www.ebi.ac.uk/QuickGO/GTerm?id=GO:0005215) |
|  | family -linked outer membrane protein | *ompA* | [structural molecule activity](http://www.ebi.ac.uk/QuickGO/GTerm?id=GO:0005198) |
|  | phosphatidylinositol alpha-mannosyltransferase | *pimA* | [phosphatidylinositol alpha-mannosyltransferase activity](http://www.ebi.ac.uk/QuickGO/GTerm?id=GO:0043750) |
|  | probable ionic transporter integral membrane protein chaa | *chaA* | [transmembrane transport](http://www.ebi.ac.uk/QuickGO/GTerm?id=GO:0055085) |
|  | maltooligosyltrehalose hydrolase | *treZ* | [trehalose biosynthetic process](http://www.ebi.ac.uk/QuickGO/GTerm?id=GO:0005992) |
|  | general substrate transporter: major facilitator superfamily protein | *SRAE_2000392300* | transmembrane transporter activit |
|  | heptose -bisphosphate phosphatase | *gmhB* | [D,D-heptose 1,7-bisphosphate phosphatase activity](http://www.ebi.ac.uk/QuickGO/GTerm?id=GO:0034200) |
|  | formate transporter | *focB* | [formatetransmembrane transporter activity](http://www.ebi.ac.uk/QuickGO/GTerm?id=GO:0015499) |
|  | dna transport machinery protein comgb | *comGB* | [protein secretion](http://www.ebi.ac.uk/QuickGO/GTerm?id=GO:0009306) |
|  | pyruvate phosphate dikinase | *ppdK* | [pyruvate, phosphate dikinase activity](http://www.ebi.ac.uk/QuickGO/GTerm?id=GO:0050242) |
|  | pts system fructose-specific transporter subunit iibc | *fruA* | [protein-N(PI)-phosphohistidine-fructose phosphotransferase system transporter activity](http://www.ebi.ac.uk/QuickGO/GTerm?id=GO:0022877) |
|  | phosphoribosediphosphate:decaprenyl-phosphatephosphoribosyltransferase | *Rv3806c* | [arabinosyltransferase activity](http://www.ebi.ac.uk/QuickGO/GTerm?id=GO:0052636) |
|  | p-type atpase-metal cation transport | *copA* | [cation-transporting ATPase activity](http://www.ebi.ac.uk/QuickGO/GTerm?id=GO:0019829) |
|  | nad+ dependent glycerol-3-phosphate dehydrogenase | *gpsA* | [glycerol-3-phosphate dehydrogenase [NAD(P)+] activity](http://www.ebi.ac.uk/QuickGO/GTerm?id=GO:0047952) |
|  | methionine transport atp-binding protein | *metN* | [ATP-binding cassette (ABC) transporter comple](http://www.ebi.ac.uk/QuickGO/GTerm?id=GO:0043190) |
|  | permease of the major facilitator superfamily oxalate formateantiporter | *BRADO5836* | [transmembrane transport](http://www.ebi.ac.uk/QuickGO/GTerm?id=GO:0055085) |
|  | stress responsive a b barrel domain protein | *ADICEAN_00201* | Stress responsive A/B Barrel Domain protein |
|  | protein stimulating phenylphosphatesynthetase activity | *ppsC* | Protein stimulating phenylphosphatesynthetase activity |
|  | solute-binding protein of transport system | *RD1_1052* | [carbohydrate transport](http://www.ebi.ac.uk/QuickGO/GTerm?id=GO:0008643) |
|  | imidazoleglycerol-phosphate dehydratase | *HISN5A* | [imidazoleglycerol-phosphate dehydratase activity](http://www.ebi.ac.uk/QuickGO/GTerm?id=GO:0004424) |
|  | heme response regulator | *hssR* | Two-component signal transduction system (phosphorelay) |
|  | fe(3+) dicitrate transport system permease protein fecd | *fecD* | [transporter activity](http://www.ebi.ac.uk/QuickGO/GTerm?id=GO:0005215) |
|  | fused histidine kinase response regulator | *PssSM_1624* | [phosphorelay sensor kinase activity](http://www.ebi.ac.uk/QuickGO/GTerm?id=GO:0000155) |
|  | phosphopantothenoylcysteine decarboxylase phosphopantothenate-cysteine ligase | *coaBC* | [phosphopantothenate-cysteine ligase activity](http://www.ebi.ac.uk/QuickGO/GTerm?id=GO:0004632) |
|  | xenobiotic-transporting atpase | *Dde_0722* | [xenobiotic-transporting ATPase activity](http://www.ebi.ac.uk/QuickGO/GTerm?id=GO:0008559) |
