# Supplementary material for: De novo transcriptome analysis of halotolerant bacterium Staphylococcus sp. strain P-TSB-70 isolated from East coast of India: In search of salt stress tolerant genes
Source: PLoS One. 2020 Feb 10;15(2):e0228199. doi: 10.1371/journal.pone.0228199 (PMC7010390; doi:10.1371/journal.pone.0228199)
Supplement: S10 Table — (DOCX) [file pone.0228199.s017.docx]

**S10 Table. SSR mining statistics in control and treated sample**

| **Description** | **Control** | **Treated** |
| --- | --- | --- |
| Total number of transcript contigs examined | 11,796 | 10,973 |
| Total size of transcript contig examined (bp) | 2,313,992 | 2,286,521 |
| Total number of identified SSRs | 102 | 102 |
| Number of transcript contigs containing SSRs | 96 | 95 |
| Number of transcript contigs containing more than one SSR | 5 | 7 |
| Number of SSRs present in compound formation | 5 | 5 |
