# Supplementary material for: De novo transcriptome analysis of halotolerant bacterium Staphylococcus sp. strain P-TSB-70 isolated from East coast of India: In search of salt stress tolerant genes
Source: PLoS One. 2020 Feb 10;15(2):e0228199. doi: 10.1371/journal.pone.0228199 (PMC7010390; doi:10.1371/journal.pone.0228199)
Supplement: S11 Table — (DOCX) [file pone.0228199.s018.docx]

**S11 Table. Distribution of SSRs in different repeat types**

| **Type of Repeat pattern** | **Control** | **Treated** |
| --- | --- | --- |
| Di-nucleotide | 84 | 86 |
| Trii-nucleotide | 0 | 1 |
| Tetra-nucleotide | 0 | 0 |
| Penta-nucleotide | 18 | 15 |
| Hexa-nucleotide | 0 | 0 |
